# Genetic basis of biofilm formation and salt adaptation in the plant-beneficial strain *Stutzerimonas stutzeri* MJL19

**DOI:** 10.1007/s00253-025-13523-0

**Published:** 2025-05-30

**Authors:** Verónica Pérez-Padilla, María Antonia Molina-Henares, Zulema Udaondo, María Isabel Ramos-González, Manuel Espinosa-Urgel

**Affiliations:** 1 Department of Biotechnology and Environmental Protection, Estación Experimental del Zaidín, CSIC, Profesor Albareda, 1, 18008 Granada, Spain; 2 Present Address: Centro Nacional de Biotecnología, CSIC, Darwin, 3, 28049 Madrid, Spain

**Keywords:** Exopolysaccharides, C-di-GMP, Salinity, Biofilm, Expression

## Abstract

**Abstract:**

*Stutzerimonas stutzeri* MJL19 represents a potential candidate for agrobiotechnological applications in regions affected by soil salinization, given its protective effects on plants under saline stress. This strain forms biofilms on some abiotic surfaces and on plant roots, a trait that influences the colonization and persistence capacities of bacteria in the rhizosphere. However, the mechanistic basis for the multicellular lifestyle of *S. stutzeri* MJL19 and its connection with the adaptation to saline conditions had not been explored. Analysis of the genome of MJL19 has allowed the identification of two gene clusters involved in the synthesis of exopolysaccharides (cellulose and a species-specific polymer). Deletion of either or both gene clusters exposed their differential roles on abiotic and biotic surfaces and phenotypic changes in response to increasing salt concentrations. Expression of both clusters is regulated by the two-component system GacS/GacA, as evidenced by analysis of a *gacS* mutant obtained by random transposon mutagenesis. This mutant also shows altered levels of the intracellular second messenger cyclic diguanylate (c-di-GMP), which is key in the transition between free-living and sessile lifestyles. Results also suggest the existence of regulatory interconnections between exopolysaccharide synthesis genes, and of these with c-di-GMP turnover, which is in turn modulated by the presence of NaCl. GacS is required for this response to varying salt concentrations. We also describe two additional elements that influence c-di-GMP levels and the response to salt: the gene *katE*, encoding catalase HP-II, and a gene that encodes a protein of the lipoteichoic acid synthases family.

**Key points:**

• GacS controls c-di-GMP levels and EPS synthesis in *S. stutzeri* MJL19 in response to salt.

• Regulation of EPS genes is interconnected and linked to c-di-GMP turnover.

• The catalase KatE influences c-di-GMP levels.

**Supplementary Information:**

The online version contains supplementary material available at 10.1007/s00253-025-13523-0.

## Introduction

*Stutzerimonas stutzeri* (formerly *Pseudomonas stutzeri*, but recently reclassified into a different genus; Lalucat et al. 2022) is a gram-negative bacterium that presents a noteworthy metabolic versatility and adaptability to different environments. This species shows significant genetic diversity, and multiple genomic groups (genomovars) have been identified (Lalucat et al. 2006). Strains have been isolated from marine samples, silver mines, contaminated soils and the rhizosphere of different plants, and on rare occasions as opportunistic infection agents (Lalucat et al. 2006). Lately, *S. stutzeri* has gained increasing attention as potential inoculant in agricultural applications due to its diazotrophic nature and capacity to stimulate plant growth, not only through nitrogen fixation but also via other mechanisms (Ke et al. 2019; Pham et al. 2017). Like many bacteria, *S. stutzeri* can form biofilms, multicellular communities embedded in self-produced extracellular polymeric substances. Biofilm formation has been reported to allow *S. stutzeri* A1501 to fix nitrogen under aerobic conditions, suggesting it is an important process contributing to its fitness in the rhizosphere (Wang et al. 2017). However, the structural and regulatory mechanisms involved in adhesion to surfaces and biofilm development by strains of this species have only recently started to be investigated in detail (Ding et al. 2019; Shang et al. 2021; Shao et al. 2022).

Strain *S. stutzeri* MJL19 was isolated from the rhizosphere of plants growing in a salt flat in Argentina and has been shown to efficiently colonize the seeds and roots of soybean (*Glycine max* L.), stimulating germination and plant growth, particularly under saline stress (Lami et al. 2020). This makes MJL19 a strain of interest from an agrobiotechnological point of view. Biochemical evidences and data from shotgun sequencing and partial assembly of the genome of MJL19 indicated the presence of functions related to plant growth promotion and protection activities, such as inorganic phosphatases or trehalose synthases (Lami et al. 2020). Genes potentially involved in root colonization and biofilm formation were also identified, and preliminary results on the influence of salinity on the sessile life of *S. stutzeri* MJL19 were obtained. These data indicated that increasing concentrations of NaCl resulted in altered colony morphology and increased biofilm formation. Such changes were accompanied by an apparent increase in the levels of the intracellular second messenger cyclic diguanylate (c-di-GMP) (Lami et al. 2020). However, these observations were not pursued in detail, and the incomplete assembly of the genome precluded a more complete analysis and further experimental approaches to delve into these findings.

Since its discovery as an allosteric effector of the enzyme cellulose synthase, c-di-GMP has acquired increasing relevance and is now recognized as a central element modulating bacterial physiology and adaptation to diverse environments (Hengge 2009; Khan et al. 2023; Liu et al. 2024; Römling 2023). It is an ubiquitous second messenger in bacteria, playing a pivotal role in many cellular processes, particularly in the transition from free-living, unicellular life to multicellular association in the form of biofilms. The biosynthesis and degradation of c-di-GMP are mediated by diguanylate cyclases (DGCs) and phosphodiesterases (PDEs), and involve active GGDEF and EAL or HD-GYP motifs, respectively; high levels of c-di-GMP generally promote the production of adhesins and extracellular polymeric substances such as exopolysaccharides (EPS), whereas low levels stimulate flagellar synthesis and motility (Hengge 2009; Jenal et al. 2017; Poulin and Kuperman 2021).

The importance of c-di-GMP and biofilm formation in plant-bacterial interactions has been documented in different systems (Huang et al. 2024; Isenberg and Mandel 2024) and could be key as a survival mechanism for bacteria and as an element of protection for plants in saline environments, given the contribution of EPS to stress tolerance and water retention (Mukherjee et al. 2019; Sharma et al. 2023). In this work, we have begun to explore the genetic elements involved in extracellular matrix production and biofilm formation by *S. stutzeri* MJL19, and their connection with c-di-GMP regulation and the adaptation to different salt concentrations.

## Material and methods

### Bacterial strains, culture media, and growth conditions

The bacterial strains and plasmids used in this study are listed in Table 1 and are available from authors upon request. *Stutzerimonas stutzeri* strains were grown at 30 °C under orbital shaking (200 rpm, unless otherwise indicated) either in Luria–Bertani (LB) medium or in M9 minimal medium (Sambrook et al. 1989) supplemented with Fe-citrate 6 μg/l, MgSO_4_ 1 mM, trace metals (final concentrations, in microgram per liter: HBO_3_, 75; ZnCl_2_, 12.5; MnCl_2_, 7.5; CoCl_2_, 50; CuCl_2_, 2.5; NiCl_2_, 5; NaMoO_4_, 7.5), as described (Yousef-Coronado et al. 2008) and with glucose (20 mM) or sodium citrate (15 mM) as alternative carbon source. *E. coli* strains were grown at 37 °C in LB under orbital shaking (200 rpm). Where indicated, LB preparation was modified to contain different final NaCl concentrations, from 0 to 500 mM (regular LB contains 170 mM NaCl). When appropriate, antibiotics were added at the following final concentrations (μg ml^−1^): ampicillin (Ap), 100; chloramphenicol (Cm), 30; gentamicin (Gm), 15 or 25, depending on the antibiotic resistance being encoded in the chromosome or harbored by a plasmid; kanamycin (Km), 25 or 50; streptomycin (Sm), 50 or 100; and spectinomycin (Sp), 50 or 100. A combination of Sm (100 μg ml^−1^) and Sp (25 μg ml^−1^) was used for selection of mutants generated by transposon mutagenesis with miniTn*5*[Sm/Sp] (see sections below).

**Table 1:** Bacterial strains and plasmids used in this work

Strain/plasmid	Relevant characteristics^a^	Reference/source
*E. coli*		
DH5α	*supE44 lacU169* (φ80*lac*ZΔM15) *hsdR17* (r_K_^−^ m_K_^−^) *recA1 endA1 gyrA96 thi-1 relA1*; cloning host	Hanahan 1985
CC118 λ*pir*	Ap^r^, Km^r^, Rif^r^; Δ (*ara-leu) araD*Δ*lacX74 galE galK phoA20 thi-1 rpsE rpoB argE* (Am) *recA1* Tn*7 λpir*. Host for mini-Tn*5* vectors	Herrero et al. 1990
S17-1 λ*pir*	λ-*pir* lysogen of S17-1 (*thi pro hsdR*^*−*^* hsdM*^+^*recA* RP4 2-Tc::Mu-Km::Tn*7*(Tp^r^ Sm^r^)). Host for miniTn*7* vectors	de Lorenzo et al. 1990
*S. stutzeri*		
MJL19	Wild-type strain	Lami et al. 2020
*gacS::Km*	Km^r^; mini-Tn*5*[Km1] transposon insertion mutant in *gacS*	This work
Δ*sea*	Km^r^; *sea* null mutant (complete operon deletion)	This work
Δ*bcs*	*bcs* null mutant (complete operon deletion)	This work
Δ*sea-Δbcs*	Km^r^; double mutant (*sea* and *bcs* deletion)	This work
Δ*sea-katE::Sm*	Km^r^; Sm^r^; Sp^r^; Δ*sea* with mini-Tn*5*[Sm/Sp] transposon insertion in *katE*	This work
Δ*sea-lsfS::Sm*	Km^r^; Sm^r^; Sp^r^; Δ*sea* with mini-Tn*5*[Sm/Sp] transposon insertion in *lsfS*	This work
*Plasmids*		
p34S-Km3	Km^r^ cassette flanked by duplicated restriction sites	Dennis & Zylstra 1998
pBBR1MCS-3	Tc^r^; broad host range mobilizable cloning vector; *lacZ*	Kovach et al. 1995
pCdrA-oriT	Ap^r^, Gm^r^; FleQ dependent c-di-GMP biosensor; mobilizable plasmid derived from pCdrA::*gfp*^*C*^	Rybtke et al. 2012; this work
pCR-Blunt II-TOPO	Km^r^; PCR cloning vector, *lacZ* α-complementation	Invitrogen
pJB3 Tc20	Tc^r^; broad host range mobilizable cloning vector	Blatny et al. 1997
pME9407	Gm^R^; miniTn*7*P_*tac*_-mCherry (constitutive expression of mCherry)	Rochat et al. 2010
pKNG101	Sm^r^; *oriR6 K sacBR*, mobilizable suicide vector	Kaniga et al. 1991
pRK600	Helper plasmid used for conjugation; *oriV*(ColE1), RK2(*mob*^+^ *tra*^+^*)*	Herrero et al. 1990
pUTmini-Tn*5*[Km1]	Km^r^; vector carrying mini-Tn*5*[Km1] derivative	de Lorenzo et al. 1990
pUTmini-Tn*5*[Sm/Sp]	Sm^r^, Sp^r^; vector carrying mini-Tn*5*[Sm/Sp] derivative	
pUX-BF13	Ap^r^; *mob*^+^; R6 K replicon-based helper plasmid, providing the Tn*7* transposase in* trans*	Bao et al. 1991
pCR-Blunt-*gacS*	Km^r^; pCR-Blunt II-TOPO with a 2759 kb PCR fragment containing *gacS*	This work
pCR-Blunt-*sea*	Km^r^; pCR-Blunt II-TOPO with a 2060 kb PCR fragment harbouring *sea* upstream and downstream regions	This work
pCR-Blunt-*bcs*	Km^r^; pCR-Blunt II-TOPO with a 1951 kb PCR fragment harbouring *bcs* upstream and downstream regions	This work
pCR-Blunt-*katE*	Km^r^; pCR-Blunt II-TOPO with a 2457 kb PCR fragment containing *katE*	This work
pCR-Blunt-*lsfS*	Km^r^; pCR-Blunt II-TOPO with a 2143 kb PCR fragment containing *lsfS*	This work
pJB3 Tc20-*gacS*	Tc^r^; 2.8 kb *Bam*HI-*Xba*I fragment of pCR-Blunt-*gacS* cloned into same sites of pJB3 Tc20, for *gacS* complementation	This work
pKNG-*sea*	Sm^r^; 2.1 kb *Not*I fragment of pCR-Blunt-*sea* cloned into same site of pKNG101	This work
pKNG-*sea:Km*	Sm^r^ Km^r^; 1 kb *Hin*dIII fragment containing the Km^r^ cassette of p34S-Km3 cloned into the same site of pKNG-*sea*, for *sea* null allele replacement	This work
pKNG-*bcs*	Sm^r^; 2 kb *Not*I fragment of pCR-Blunt-*bcs* cloned into the same site of pKNG101, for *bcs* null allele replacement	This work
pBBR-*katE*	Tc^r^; 2.5 kb *Xma*I fragment of pCR-Blunt-*katE* cloned into the same site of pBBR1MCS-3, for *katE* complementation	This work
pBBR-*lsfS*	Tc^r^; 2.1 kb *Spe*I-*Xba*I fragment of pCR-Blunt-*lsfS* cloned into the same sites of pBBR1MCS-3, for *lsfS* complementation	This work

^a^*Ap*, ampicillin; *Cm*, chloramphenicol; *Gm*, gentamicin; *Km*, kanamycin; *Rif*, rifampicin; *Sm*, streptomycin; *Sp*, spectinomycin; *Tp*, trimethoprim

### Molecular biology techniques

Restriction enzyme digestion and electrophoresis were carried out using standard methods (Sambrook et al. 1989) according to the manufacturers’ instructions (Roche, New England BioLabs and Canvax). Total DNA extraction was performed using the Wizard® genomic DNA purification kit (Promega). Plasmid DNA isolation and recovery of DNA fragments from agarose gels were done using Qiagen miniprep and gel extraction kits respectively. Transformation assays were conducted using established protocols (Sambrook et al. 1989) using *E. coli* DH5α as host strain (Hanahan 1985) unless otherwise indicated. Electrotransformation of *S. stutzeri* was performed as previously described for *Pseudomonas* (Enderle and Farwell 1998) using an EC100 electroporator according to the manufacturer’s guidelines (EC Apparatus Corporation). PCR amplification was done using Q5® High-Fidelity DNA Polymerase (New England BioLabs). The absence of missense mutations and integrity of the PCR amplicons was verified by sequencing, carried out at the Genomics Service, IPBLN-CSIC (Granada, Spain).

### Genome sequencing and assembly

One microgram DNA obtained as described above was used for re-sequencing of the genome of *S. stutzeri* MJL19. Sequencing was done at FISABIO (Valencia, Spain) using Pac-Bio, with an estimated coverage of 260 × (394,400 reads, corresponding to over 1.66 × 10^9^ bases). These sequencing results and the previously published shotgun sequencing data obtained with Illumina (36 contigs; Lami et al. 2020) were used in a hybrid assembling approach to obtain the complete genome assembly of MJL19 using Unicycler v0.5.1 assembler (Wick et al. 2017) The pipeline resulted in a single circular chromosome of 4.57 Mbp containing 4,277 genes. A quantitative genome assembly assessment was performed using ChecK v1.2.1 (Parks et al. 2015), provided a completeness score of 99.52%. Gene calling and annotation of the resulting complete genome was performed using the NCBI Prokaryotic Genome Annotation Pipeline v6.6 (Tatusova et al. 2016). Data have been deposited in GenBank (accession CP140298.1).

### Conjugation

Plasmid transfer from *E. coli* to *S. stutzeri* MJL19 and its derivatives was performed through biparental, triparental, or tetraparental matings as required, using as donors *E. coli* strains harboring the plasmids indicated in each case for random transposon mutagenesis, fluorescent tagging, or allelic replacement (Table 1). *Escherichia coli* strains harboring pRK600 or pUX-BF13 (Herrero et al. 1990; Bao et al. 1991) were used as helper strains, as required in each case. Overnight LB cultures of donor, helper, and recipient bacteria were mixed in a 1:1:1 proportion, followed by centrifugation at 13,000 rpm for 1 min. The pellet was washed twice with 0.5 ml fresh LB medium to remove any residual antibiotics and subsequently resuspended in 50 μL of LB medium. This bacterial suspension was then spotted onto a sterile nitrocellulose filter (0.22 μm), which was placed on an LB agar plate and incubated for 6 h at 30 °C. Cells were then scraped off the filter, suspended in 1 ml of M9 salts, and plated on M9 with 15 mM sodium citrate and appropriate antibiotics, to select transconjugants and counterselect *E. coli* strains. Longer incubation times of the mating mix resulted in higher aggregation of *S. stutzeri* that hampered later resuspension, while conjugation efficiency was not noticeably increased.

### Random transposon mutagenesis and selection of mutants

Transposon mutagenesis with miniTn*5*[Km1] or miniTn*5*[Sm/Sp] was performed by triparental conjugation (de Lorenzo et al. 1990; Herrero et al. 1990) with either MJL19 or its Δ*sea* derivative as the recipient strain. The first was used to select mutants exhibiting altered colony morphology on plates. The second was employed to identify mutants with altered levels of c-di-GMP. For that purpose, clones obtained after random transposon mutagenesis were collected, and this pool was used to introduce the pCdrA-oriT biosensor (see below) by conjugation. The resulting colonies were analyzed using a Leica M165 FC epifluorescence stereomicroscope to identify changes in fluorescence emission. The identity of *S. stutzeri* derivatives and the presence of the transposon was checked by PCR amplificaction with appropriate primers (fps158 + rps743; Km1 Fw + Km1Rev; miniTn5-SmF + miniTn5-SmR; see Table S1 for details).

### Identification of miniTn5 insertion sites

Transposon insertion sites were determined by arbitrary PCR, followed by sequencing, similarly to the method described previously (Espinosa-Urgel et al. 2000), with some modifications to optimize it for *S. stutzeri*. Primers used are listed in Table S1. A first round of amplification was done using as template chromosomal DNA of the mutants digested with *Sal*I, which does not disrupt the inserted sequence, and a mix of two arbitrary primers containing a random sequence and a fixed one (ARB1A and ARB1D) and an internal primer corresponding to a sequence close to the I-end of miniTn*5* (TNEXT2 for miniTn*5*[km1] and TNTETU for miniTn*5*[Sm/Sp]). A second round of amplification was done with 5 μl of the first-round reaction as the template and a mix of two primers corresponding to the fixed sequence of the first pair of arbitrary primers (ARB2A and ARB2D) and a second internal primer of miniTn*5*, closer to the I-end (TNINT or TNINT7 for miniTn*5*[km1] and TNTCO or TNINT-Sm for miniTn*5*[Sm/Sp]). PCR products were electrophoresed, the most intense bands were isolated with QIAquick® Gel Extraction Kit (QIAGEN) and sequenced at the Genomics Service of the IPBLN-CSIC. The obtained sequences were compared with the genome sequence of MJL19 using BLAST programs (http://www.ncbi.nlm.nih.gov/blast/Blast.cgi).

### Confirmation of miniTn7 insertion

Analysis of the complete genome sequence of MJL19 allowed identifying a single recognition site (*att*Tn*7*) for miniTn*7* (5’-CCAGCCGCGCAACCTGGCCAAGTCGGTTAC-3’) (Zhang et al. 2021), leading to insertion in the extragenic region downstream *glmS*. Oligonucleotides Tn7RR109 and P.s.glmS3’ annealing respectively at the end of Tn*7*R and at the 5’ extreme of the *glmS* gene of MJL19 were used to confirm the legitimate insertion of miniTn*7*.

### Generation of null mutants and complemented strains

Mutants were generated by full deletion of the corresponding gene(s) through double homologous recombination, using a methodology similar to that previously detailed (Tagua et al. 2022). Upstream and downstream fragments surrounding the region to be deleted were amplified by overlapping PCR using Phusion High-Fidelity DNA polymerase (Thermo Fisher Scientific). Oligonucleotides used are detailed in Table S1. The amplicons were cloned into pCR™-Blunt II-TOPO®. The absence of missense mutations in the cloned fragments was verified by sequencing. Subsequently, these fragments were integrated in pKNG101 (Kaniga et al. 1991). Where indicated, a Km resistance cassette from plasmid pS34 Km (Dennis and Zylstra 1998) was incorporated to facilitate selection of mutants. The resulting plasmids (see Table 1 for full details) were transferred to *S. stutzeri* MJL19 by triparental conjugation, and clones where a double recombination event had taken place were identified through initial selection on M9-citrate plates with Sm, followed by growth in the presence of 10% sucrose and confirmation of Sm sensitivity. The presence of null mutations was checked by PCR with internal primers (Table S1).

For complementation studies, the complete open reading frames of *gacS*, *katE*, and *lsfS* were amplified by PCR using the primer pairs indicated in Table S1. The amplified fragments extended to the corresponding non-coding upstream regions (90, 271, and 141 bp, respectively) in order to include promoter sequences. The PCR products were digested with *Bam*HI and *Xba*I (New England Biolabs) and introduced into pJB3 Tc20 (Blatny et al. 1997) to construct plasmid pJB-*gacS*, or with *Spe*I and *Xma*I (New England Biolabs) and introduced into pBBR1MCS-3 (Kovach et al. 1995) to construct plasmids pBB-*kat*E and pBB-*lsfS*. All inserts were sequenced to ensure the lack of mutations.

### Quantitative real-time PCR

Biological duplicates of bacterial cultures were grown overnight on LB medium. Cells were recovered by centrifugation, immediately frozen in liquid nitrogen and stored at −80 °C. RNA extraction of three technical samples per biological replicate of each strain and quantitative real-time PCR (qRT-PCR) were performed using Sybr Green mix (Molecular Probes), as described previously (Molina-Henares et al. 2024). Template cDNAs from the three technical samples were amplified using the primers listed in Table S1, designed to produce PCR products ranging from 125 to 220 bp. Gene expression results of the target genes were normalized relative to those obtained for the 16S rRNA gene using BIO-RAD iQ5 software. Quantification was based on the 2^−ΔΔCT^ method (Livak and Schmittgen 2001).

### Generation of the conjugative biosensor pCdrA-oriT

A fragment of 779 bp containing the RP4-oriT site of pUNϕ18 (S. Marqués, personal communication) was PCR amplified with primers indicated in Table S1 and cloned into PCR TOPO™ Zero Blunt™ (Invitrogen) generating pMIR276. After discarding any alteration in the sequence, the *Bsp*EI fragment of pMIR276 with the *oriT* site was inserted at the unique *Bsp*EI site of the non-conjugative pCdrA::*gfp*^c^ (Rybtke et al. 2012), giving rise to its conjugative derivative pCdrA-oriT. Plasmid pCdrA-oriT was introduced in *S. stutzeri* strains by conjugation, for observation and quantification of c-di-GMP by fluorescence emission.

### Biosensor-based c-di-GMP visualization and quantification

Overnight cultures grown in liquid media were diluted to an optical density at 660 nm (OD_660_) of 1, and 2 μL of the suspensions were spotted onto LB plates, allowed to dry and incubated at 30 °C. At the indicated times, fluorescence was observed using a Leica M165 FC epifluorescence stereomicroscope equipped with a filter set corresponding to excitation/emission wavelengths of 460–480/515 nm.

Quantitative analysis of fluorescence was done using a Thermo Scientific™ Varioskan LUX™ Multimode Microplate Reader at an excitation/emission spectrum of 485/515 nm. Experiments were carried out with cultures grown either in solid or liquid media as follows. For cultures in solid medium, 96-wells black microplates were used, each well containing 200 μl of LB agar or M9 agar minimal medium with glucose. Overnight bacterial cultures grown in liquid media were diluted to an OD_660_ of 0.05 and 20 μl of the bacterial suspension were added to each well. The cultures were subsequently incubated at 30 °C and fluorescence was measured after 24 h. In the case of liquid cultures, kinetic studies were carried out using transparent-bottom black microplates containing 200 μl of liquid M9 minimal medium. Plates were inoculated as above, with at least five replicas per strain and/or experimental condition. To minimize experimental variability due to evaporation, peripheral rows and columns of the plates were left unfilled. Turbidity (OD_660_) and fluorescence were recorded every 30 min during growth at 30 °C under static conditions, punctuated by intermittent shaking for 10 s before each measurement. Relative fluorescence units (RFUs) were computed by normalizing the raw fluorescence data to the turbidity at each timepoint, thereby adjusting fluorescence readings relative to bacterial cell growth.

### Quantitative analysis of c-di-GMP by LC–MS/MS

Extraction of c-di-GMP was conducted following a modified protocol originally provided by Biolog (Germany), tailored for bacterial liquid cultures. Bacterial cultures were grown overnight in liquid M9 minimal medium supplied with 20 mM glucose as a carbon source. Cultures were then adjusted to an optical density at 600 nm of 1 in a final volume of 5 ml prior to c-di-GMP extraction. Cells were centrifuged twice before being resuspended in extraction solution (acetonitrile/methanol/water, 2/2/1, v/v/v). The suspensions were incubated for 15’ on ice, followed by 10’ incubation at 99 °C. Samples were cooled down and centrifuged. The supernatants were transferred to clean tubes, and pellets were subjected to two additional rounds of extraction. The final fluids were combined and stored overnight at − 20 °C. After a final centrifugation step to remove remaining proteins, the final extracts were evaporated to dryness in a Speed-Vac. The extraction assay for each strain was conducted in duplicate, with four independent technical repeats. Quantification of c-di-GMP concentrations was carried out using liquid chromatography-tandem mass spectrometry (LC–MS/MS) by Biolog LSI (Germany). Data were normalized with respect to colony-forming units (CFU) to estimate the concentration of second messenger per cell.

### Biofilm, motility, and Congo red binding assays

Biofilm assays were performed with bacterial strains harbouring miniTn*7*P_*tac*_-mCherry (Rochat et al. 2010) in single copy in the chromosome, which was introduced by triparental conjugation.

Overnight bacterial LB cultures were diluted to an OD_600_ of 0.05 and inoculated in triplicate in a total volume of 4 ml liquid LB medium in borosilicate tubes with a sterile toothpick inserted. Cultures were incubated at 30 °C under orbital shaking at 130 rpm. Biofilms on the glass surface were visualized and photographed, and those formed on the toothpick surfaces were observed using a Leica M165 FC epifluorescence stereomicroscope (bright field and fluorescence, with a filter set corresponding to excitation/emission wavelengths of 540–580/610 nm). Images were analyzed using ImageJ (http://imagej.nih.gov/ij).

Swimming motility assays were conducted by spotting 2 μl of overnight LB bacterial cultures, adjusted to an OD_600_ = 1 onto LB agar plates containing 0.3% agar. Migration halos were photographed after 20 h of growth at 30 °C. Surface motility was analyzed similarly, except that PG-swarming agar was used, as described (Matilla et al. 2007), and incubation was carried out at 25 °C for 72 h. Congo red binding was evaluated on modified LB plates with varying concentrations of NaCl (0 mM, 100 mM, 250 mM, and 500 mM) and 40 μg/ml Congo red. Overnight cultures were spotted as above, incubated at 30 °C for 24–48 h, and morphology and dye binding were observed using a Leica M165 FC stereomicroscope.

### Statistical analyses

The Shapiro–Wilk test was used to evaluate the assumption of normality in the datasets. Homogeneity of variances across groups was assessed using Levene’s test. One-way analysis of variance (ANOVA) followed by Tukey’s Honestly Significant Difference (HSD) post hoc test was conducted to determine statistically significant differences among group means. All statistical analyses were performed using IBM® SPSS® Statistics software.

## Results

### Complete genome assembly and re-annotation of S. stutzeri MJL19

Genomic DNA of *S. stutzeri* MJL19 was extracted and purified as described in the “Methods” section, and used for whole-genome sequencing using PacBio, at the FISABIO sequencing service (Valencia, Spain). A total of 394,400 reads were obtained, corresponding to 1.66 × 10^9^ bases (approximately 260 × coverage). These and the previously reported data, consisting of 36 contigs (Lami et al. 2020), allowed the full assembly and re-annotation of the genome of MJL19, which is available in GenBank with accession CP140298.1.

The genome of *S. stutzeri* MJL19 consists of a single chromosome with a total size of 4,572,525 base pairs (bp) and a GC content of 64%. Functional annotation of the strain identified 4277 genes, of which 4201 are protein-coding sequences. Additionally, 60 genes were identified as tRNAs, and four complete ribosomal RNA operons were found distributed throughout the chromosome (Figure S1). Sequence data indicated the presence of two flagellar systems, genes involved in type IV pili, curli and exopolysaccharide synthesis (see below), and the absence of large adhesins as those found frequently in rhizosphere-colonizing pseudomonads (Espinosa-Urgel and Ramos-González 2023).

### Identification of genetic determinants with a potential role in biofilm formation

MJL19 forms characteristic wrinkly, dry colonies, which are difficult to remove from the agar surface, and tends to aggregate and form clumps during growth in rich liquid media. In other bacteria, similar phenotypes can be observed when a diguanylate cyclase is overexpressed, leading to increased production of exopolysaccharides (Matilla et al. 2011; Xu et al. 2021; Blanco-Romero et al. 2022). Thus, our starting hypothesis was that colony morphology could be related to the high physiological levels of c-di-GMP detected in this strain (Lami et al. 2020), and mutations leading to altered morphology might reveal changes in second messenger signaling and/or structural elements of the extracellular matrix relevant for biofilm formation. As a first approach, we carried out random transposon mutagenesis on MJL19, using mini-Tn*5*[Km1], attempting to identify mutants with different colony morphologies. This technique turned out not to be very efficient in *S. stutzeri* MJL19, likely due to the triparental conjugation process being hampered by the thick extracellular material produced by this strain, and also because of the difficulties in dispersing the clumps formed during the incubation period of the mating mix for further spreading on agar plates. All this resulted in the isolation of a limited number of clones that had incorporated the transposon. After several rounds of mutagenesis, we could identify one mutant, out of around 2000 clones, that had lost the characteristic wrinkly colony morphology of MJL19 and formed smooth, nearly translucent colonies. This mutant was further analyzed by arbitrary PCR and sequencing, as indicated in the Methods section, to identify the site of insertion of mini-Tn*5*[Km1]. The transposon disrupted a 2756 bp open reading frame (U3Q39_RS07180) encoding a protein 68% identical to GacS of *Pseudomonas putida*, the sensor histidine kinase of the two-component system GacS/GacA, which has been described in different bacterial species as a master regulatory system for many processes, including biofilm formation (Lapouge et al. 2008; Martínez-Gil et al. 2014; Kim et al. 2014a). We have named the gene accordingly. Insertion of mini-Tn*5*[Km1] had taken place 625 bp downstream the ATG. This *gacS::Km* mutant was chosen for further analysis (see following sections). A GacA homolog, corresponding to locus U3Q39_RS09975, was also identified in a different region in the genome of MJL19.

Given the limited success obtained with the previous method, a targeted approach was also adopted. The genome of MJL19 was surveyed to identify potential elements involved in biofilm formation. This survey allowed the identification of two gene clusters encoding enzymes for the synthesis of EPS (Figure S2). Based on protein similarities, one of these clusters corresponds to the synthesis of bacterial cellulose and is designated *bcs*, while the second one appears to be species specific, and according to BLAST analyses of this region, the whole gene cluster is not conserved in all strains of *Stutzerimonas stutzeri* (data not shown). We refer to this cluster as *sea* (for *stutzeri* exopolisaccharide a). Mutants in each of these EPS systems and the double mutant were generated by deletion of the corresponding gene clusters (Figure S2) by means of homologous double recombination. Hereon we refer to these mutants as Δ*sea*, Δ*bcs*, and Δ*sea*-Δ*bcs*.

### Phenotypic characterization of mutant strains

**Fig. 1:**
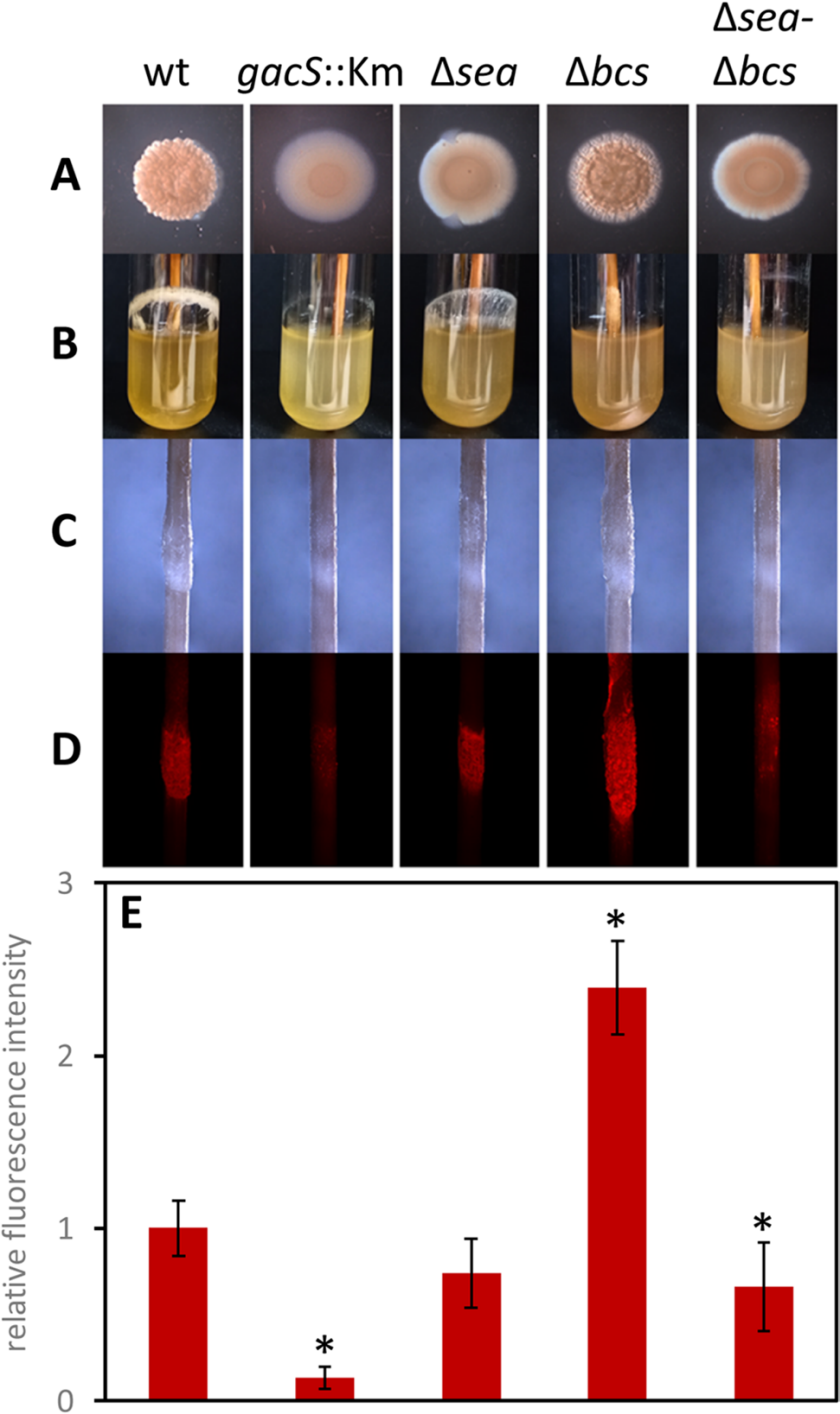
Colony morphologies and biofilm formation of wild-type MJL19 and mutant derivatives after 48 h of growth. Strains harboring miniTn*7*[P_*tac*_-mCherry] were spotted on LB plates or grown in liquid LB medium under orbital shaking (130 rpm). Colony morphologies (**A**) and biofilm formation on borosilicate glass tubes (**B**) were visualized. Biomass attached to the surface of a wooden toothpick was analyzed with an epifluorescence microscope, under brightfield (**C**) or fluorescence with appropriate filter sets (**D**). Experiments were done in triplicate and a representative image is shown. **E** Relative fluorescence intensity from images in **D**. Images were converted to 32-bit grayscale and analyzed using ImageJ. Data are averages and standard deviations of the triplicate data, normalized with respect to the wild type (relative value = 1). Statistically significant differences are indicated (*)

All the mutants grew normally in rich (LB) and defined (M9 with glucose or citrate as carbon source) media, although the typical cell clumping phenotype observed in liquid cultures after 24 h was less evident in the Δ*sea* and Δ*sea*-Δ*bcs* mutants. Also, morphological differences with the wild type could be observed during growth on LB agar, except in the Δ*bcs* mutant (Fig. 1A). These differences were less evident on minimal medium, where the wrinkly morphology of MJL19 colonies takes longer to be apparent (not shown).

To determine if these modifications correlated with changes in surface colonization, biofilm formation was analyzed during growth in liquid LB under orbital agitation in borosilicate glass tubes. A toothpick was included in each tube since we had observed in previous experiments that MJL19 tends to form a thick biofilm on the wooden surface but shows limited attachment to plastic surfaces (not shown), in contrast with *S. stutzeri* A1501, which forms biofilms on plastic microtiter plates when grown in LB (Shang et al. 2021). To facilitate visualization and allow distinguishing between cells and extracellular material on the toothpick, all strains were tagged with miniTn*7*[P_*tac*_-mCherry] in single copy in the chromosome, as described in “Materials and methods” section. Similar fluorescence was observed in all the tagged strains (not shown). Biofilms formed on the glass and wood surfaces were observed after 48 h. Biofilm formation on the glass surface was reduced in the Δ*sea* mutant and almost completely abolished in the *gacS::Km*, Δ*bcs*, and Δ*sea*-Δ*bcs* mutants (Fig. 1B). Stereomicroscopy observation of the toothpicks (Fig. 1C) indicated that the *gacS::Km* strain was also limited in terms of biomass associated to the wood surface. Intriguingly, the Δ*bcs* mutant showed similar, or even slightly increased biomass attached to the toothpick compared to the wild type, whereas a reduction was observed in the Δ*sea* and Δ*sea*-Δ*bcs* mutants. When the wood surface was analyzed using epifluorescence microscopy (Fig. 1D), reduced fluorescence was observed in the *gacS::Km* and the Δ*sea*-Δ*bcs* mutants, whereas the Δ*bcs* mutant exhibited increased fluorescence. No consistent differences with the wild type were evident in the case of the Δ*sea* mutant. These observations were confirmed by quantitative analysis using ImageJ (Fig. 1E). Observation of all the strains by transmission electron microscopy suggested the existence of differences in the amount and nature of the extracellular material in all the mutants under these growth conditions (Figure S3).

**Fig. 2:**
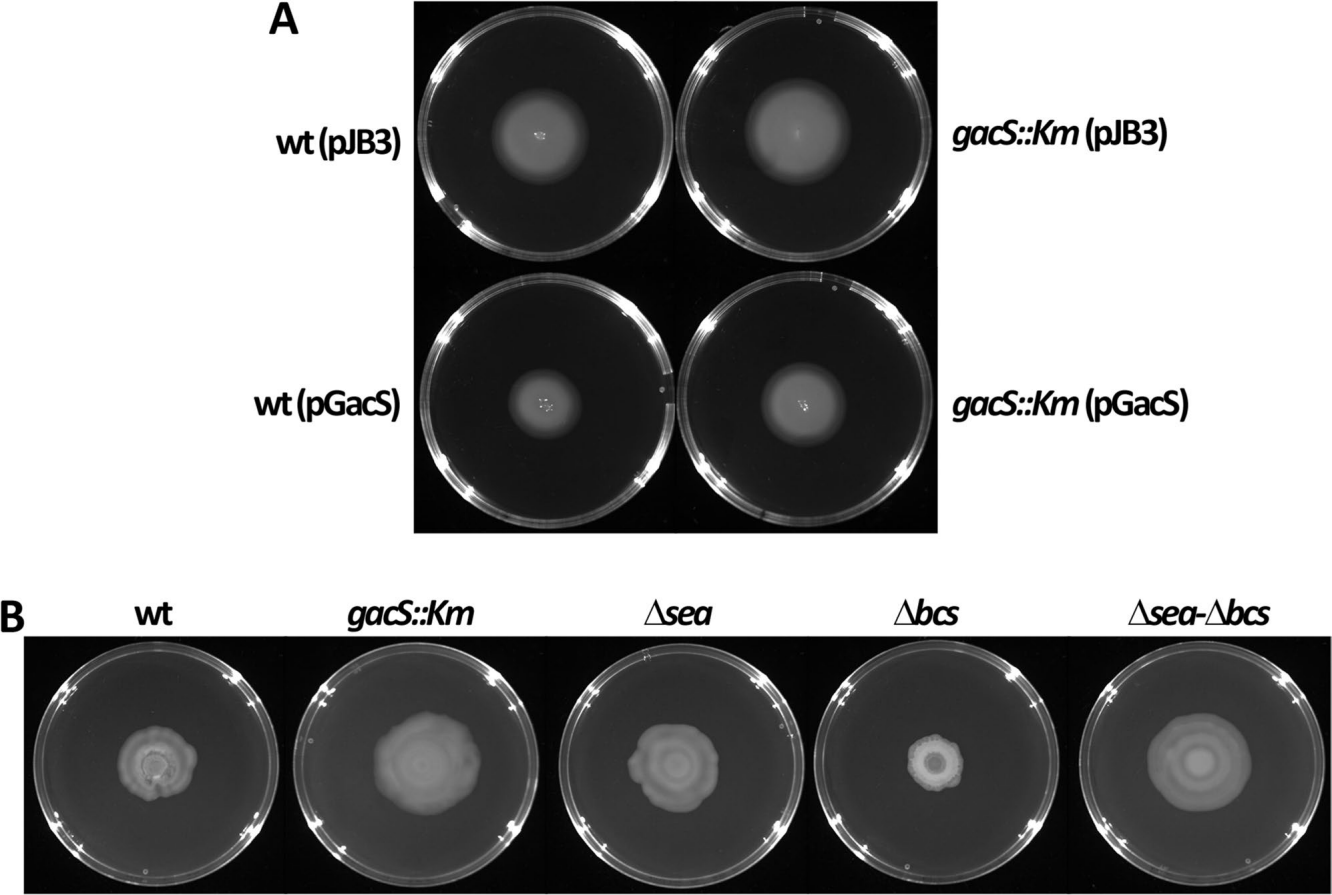
Swimming (**A**) and surface (**B**) motility of the different strains on LB or PG agar plates, after 20 and 72 h, respectively; pJB3 and pGacS are abbreviations for pJB3 Tc20 and pJB3 Tc20-*gacS* (Table 1), respectively

The two-component system GacS/GacA has been described to modulate flagellar movement in *Pseudomonas* and other bacteria (Martínez-Granero et al. 2006; Navazo et al. 2009; López-Pliego et al. 2021), and a correlation between EPS production and surface motility exists in different microorganisms (Liu et al. 2016; Yuan et al. 2022). Therefore, motility was analyzed in the various strains. The *gacS::Km* mutant showed a very slight increase in swimming motility in LB with 0.3% agar, while the wild-type *gacS* allele in multicopy caused a reduction, both in MJL19 and the *gacS::Km* mutant (Fig. 2A). These differences were nonetheless very minor, and none of the remaining mutants presented alterations in terms of swimming motility (not shown). In contrast, spread of the Δ*bcs* mutant on the agar surface was slightly reduced with respect to MJL19, whereas an increase was observed in the remaining mutants (Fig. 2B).

### Expression of EPS clusters is interconnected and regulated by GacS

**Fig. 3:**
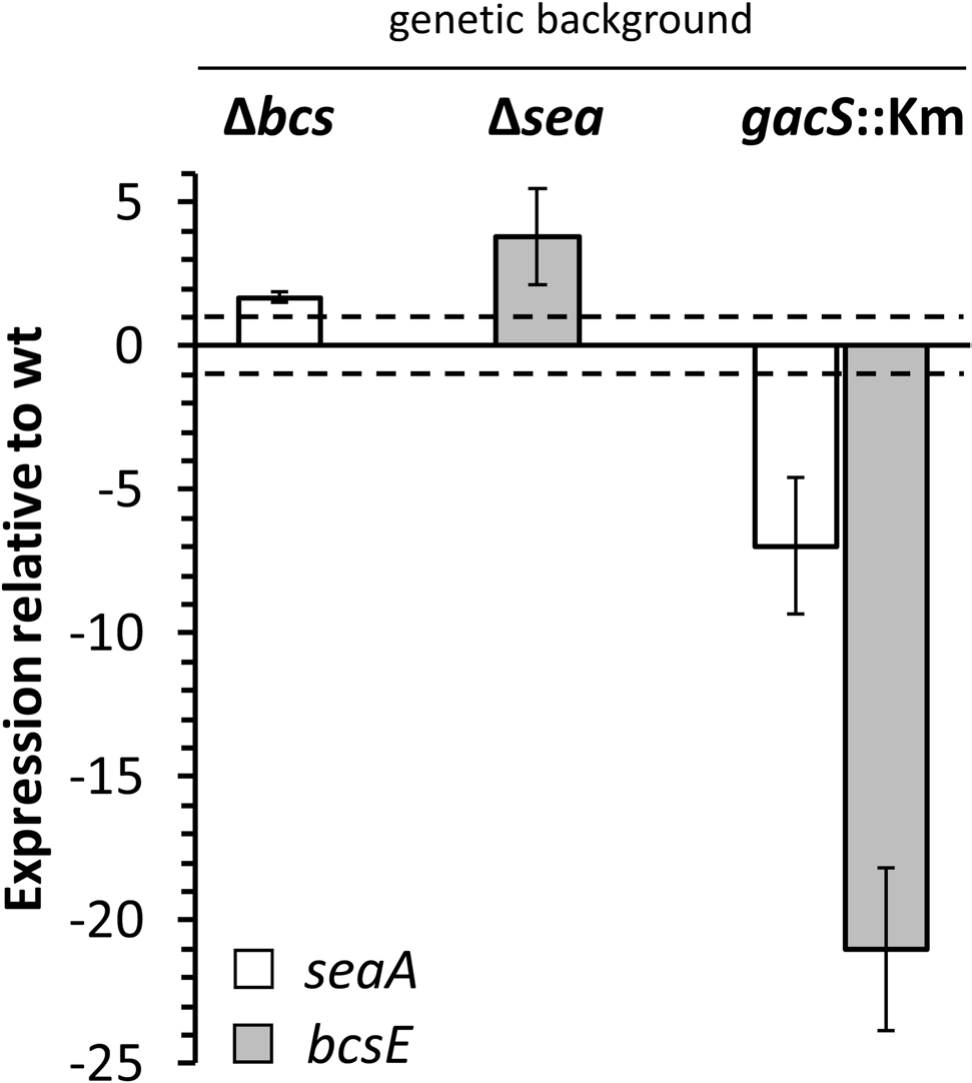
Relative expression levels of *seaA* (white bars) and *bcsE* (grey bars) in the *gacS::Km* Δ*sea*, and Δ*bcs* backgrounds compared to the wild type, analyzed by qPCR. Data are averages and standard deviations of two biological replicas with three technical replicas each. All the differences with respect to the wild type were statistically significant (Student’s *t* test; *P* < 0.05), considering a 1.5-fold change as the cutoff value (dashed lines)

The denser biofilm formed on the toothpick by the Δ*bcs* mutant could indicate either that cellulose production interferes with Sea-mediated colonization of this surface, or that the lack of cellulose causes a rise in the production of Sea, leading to increased biofilm formation on this surface. A modulation interplay between proteins and EPS components of the extracellular matrix has been observed in other bacteria (Martínez-Gil et al. 2013), a fact that led us to evaluate if expression of the *sea* and *bcs* clusters was interconnected, and to test the influence of GacS. For that purpose, quantitative real-time PCR was used to evaluate expression of the first gene of each cluster, *seaA* and *bcsE*, in cultures of the wild type and mutant strains grown in liquid LB. Results are presented in Fig. 3. A slight increase (1.7-fold) in expression of *seaA* was observed in the Δ*bcs* mutant with respect to the wild type, and a 3.8-fold increase in *bcsE* expression could be detected in the Δ*sea* mutant, suggesting that there is in fact some kind of compensating effect between the two EPS. Both genes showed a clear reduction in expression (7- and 21-fold, respectively) in the *gacS::Km* mutant (Fig. 3), indicating that the GacS/GacA two component system has a positive regulatory role in the production of both EPS.

### GacS and the species-specific EPS Sea contribute to phenotypic changes in response to salt

**Fig. 4:**
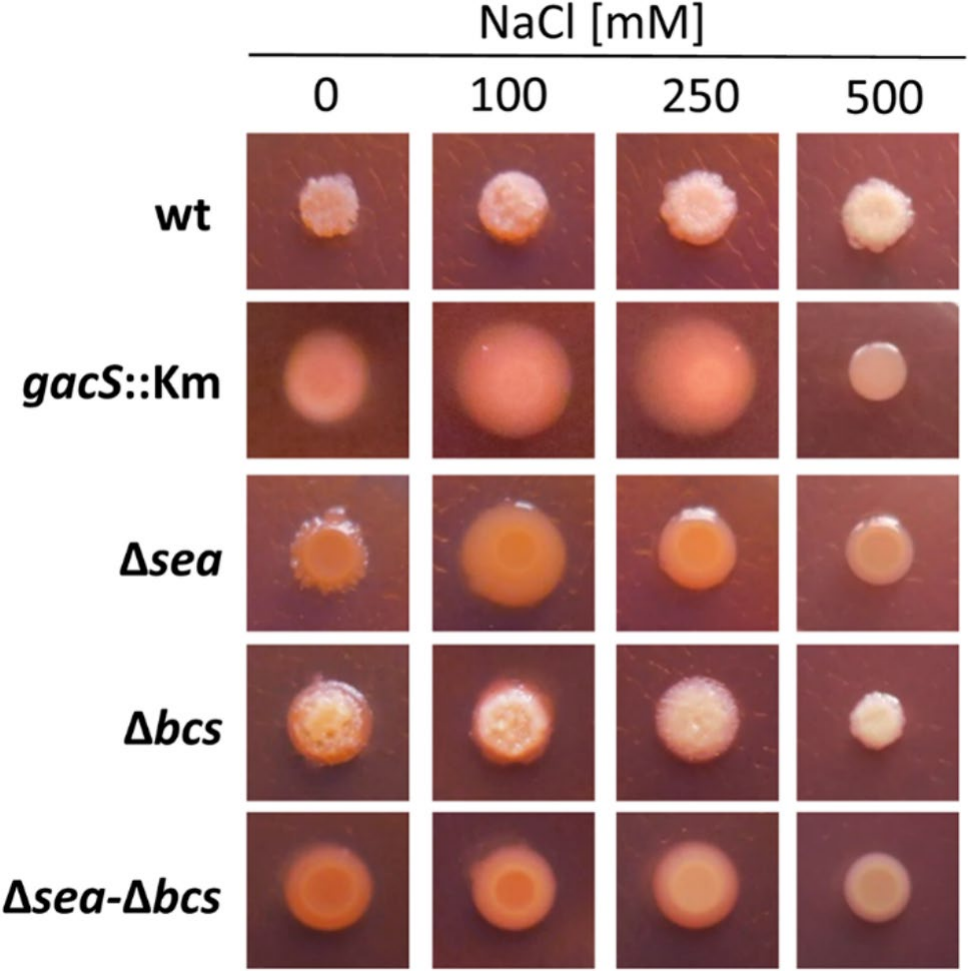
Effect of increasing concentrations of NaCl on colony morphology and Congo red binding of MJL19 and mutant derivatives. Images were taken after 24 h of growth at 30 °C

Changes in colony size and biofilm formation have been previously reported for MJL19 growing at different NaCl concentrations (Lami et al. 2020). To check if the different mutations had an influence on this response, cultures of the wild type and mutant strains were grown in liquid LB, adjusted to an OD_600_ = 1 and 5 μL were spotted onto modified LB plates, with final concentrations of NaCl 0, 100, 250, or 500 mM, and supplemented with Congo red. Color, size, and morphology of the bacterial patches were observed after 24 h of growth at 30 °C. Results are shown in Fig. 4. In general, all strains formed smaller, more compact colonies at 500 mM NaCl. The wild type and Δ*bcs* strains formed rough colonies at all concentrations, with low accumulation of the dye, although slight differences could be observed in size and color between the two strains. In the remaining mutants, the changes in morphology and color were evident, with a smooth surface and increased accumulation of Congo red, indicative of modifications in the composition of the extracellular matrix under these conditions.

### GacS regulates c-di-GMP contents in MJL19

**Fig. 5:**
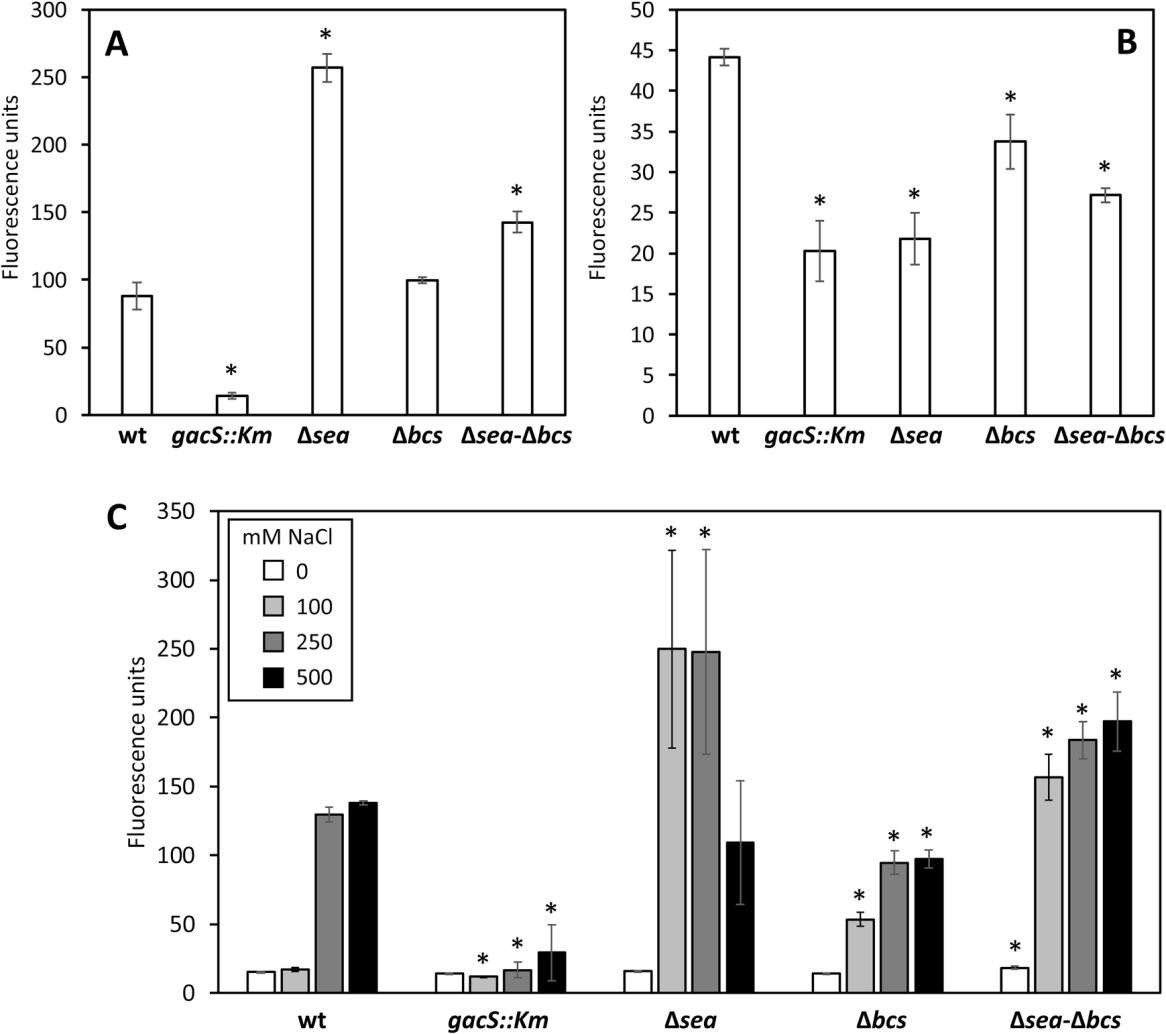
Biosensor-based analysis of c-di-GMP contents in MJL19 and its mutant derivatives harboring pCdrA-oriT, after 24 h of growth on solid media: regular LB (**A**), M9-glucose (**B**), and LB modified to contain different NaCl concentrations (**C**). Data correspond to fluorescence units measured in a Varioskan Lux plate reader and are averages and standard deviations of five independent samples per strain. Asterisks indicate statistically significant differences with respect to the wild type in each condition tested (Student’s *t* test; *P* < 0.05)

Previous evidence suggested that different NaCl concentrations could alter c-di-GMP levels in MJL19 (Lami et al. 2020), but precise quantification was not carried out. To analyze in detail these changes and determine the influence of the various mutations in c-di-GMP turnover, pCdrA-oriT, a version of the pCdrA-*gfp*^C^ biosensor (Rybtke et al. 2012) modified to be conjugative (see Methods section) was introduced in all the strains and fluorescence was evaluated in different conditions in 96-well plates, using a Varioskan Lux plate reader. Initially, overnight cultures were spotted on solid media (LB or M9 with glucose as carbon source) and allowed to grow at 30 °C, and fluorescence was measured after 24 h. Results are shown in Fig. 5A and B and indicate a relevant role of GacS, with a significant reduction in c-di-GMP levels in the *gacS::Km* mutant, which is more evident in LB than in minimal medium. Interestingly, changes in c-di-GMP levels were also observed in the Δ*sea* and Δ*sea*-Δ*bcs* mutants, but these show opposite trends in the two growth media with increased fluorescence in LB and decreased in M9-glucose. Fluorescence was not altered in the Δ*bcs* mutant in LB but a reduction was observed in minimal medium.

To explore in more depth these results and evaluate the influence of salt, a similar experiment was carried out in LB agar with different NaCl concentrations. Results are presented in Fig. 5C. As expected from previous qualitative data (Lami et al. 2020), fluorescence of the wild-type strain harboring pCdrA-oriT increased in response to high salinity (250 and 500 mM NaCl). This response was almost completely abolished in the *gacS::Km* mutant, which showed lower fluorescence than the wild type, except in the absence of salt, where values were very similar. In contrast, the mutant devoid of both EPS presented higher fluorescence than the parental strain at all the NaCl concentrations tested. The single mutants in either EPS retained the response to salt, but with different profiles: the Δ*bcs* strain showed increased fluorescence at 100 mM with respect to the wild type, and slightly reduced fluorescence at higher concentrations; the Δ*sea* mutant showed increased fluorescence at the intermediate concentrations (100 and 250 mM NaCl), but there was a large variability among samples. These data indicate that GacS is required for the response of *S. stutzeri* MJL19 to increasing salinity in terms of c-di-GMP signaling, and that production of the components of the extracellular matrix is somehow monitored to modulate c-di-GMP synthesis.

**Fig. 6:**
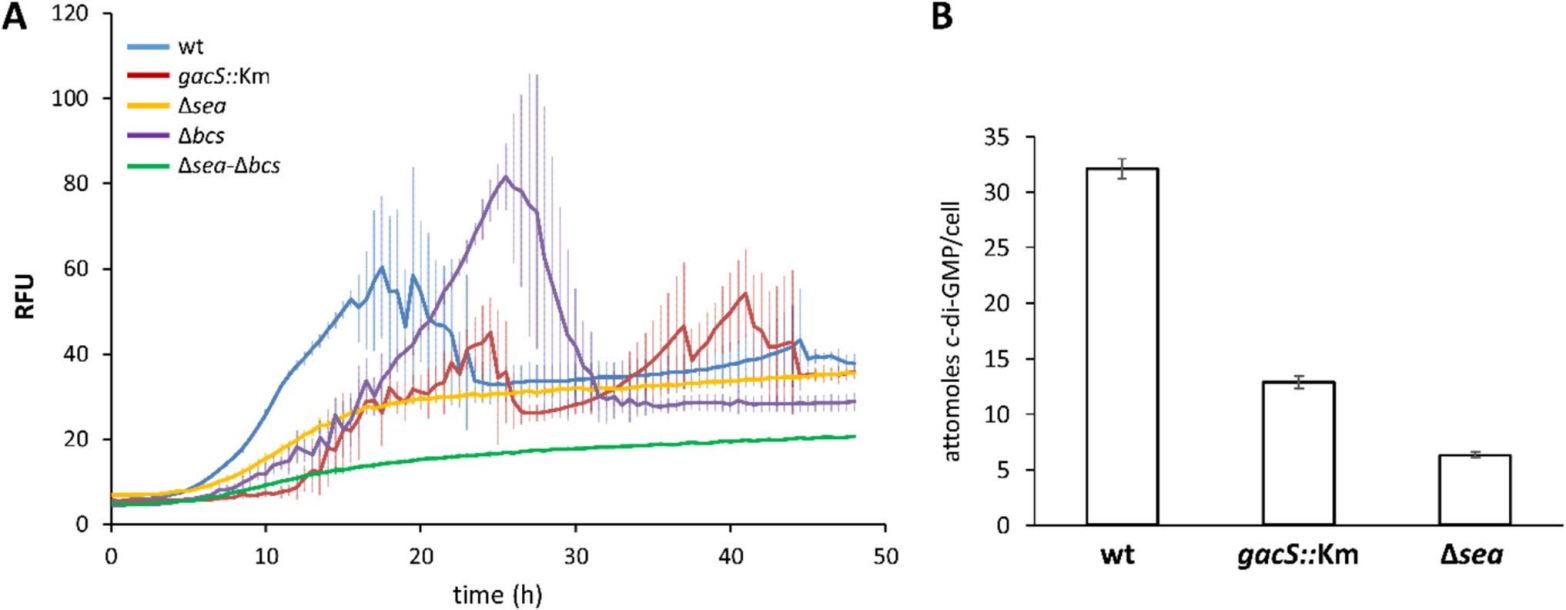
**A** Biosensor-based analysis of c-di-GMP contents in MJL19 (blue line) and its mutant derivatives *gacS::Km* (red), Δ*sea* (yellow), Δ*bcs* (purple), and Δ*sea*-Δ*bcs* (green) during growth in liquid M9-glucose medium at 30 °C, measured in a Varioskan Lux plate reader. Fluorescence and turbidity were measured every 30’, and relative fluorescence was calculated. Values are averages and standard deviations (vertical lines) of five independent samples per strain. **B** LC–MS analysis of c-di-GMP levels in MJL19 and mutant derivatives grown o/n under shaking in minimal medium with glucose as carbon source. Data are averages and standard deviations of two biological replicas with four technical repeats

The above setup using solid media has the advantage of evaluating c-di-GMP contents in surface-associated bacterial populations, as a proxy of what may take place in root-attached cells, but it does not allow for normalization of fluorescence with respect to bacterial density. On the other hand, the aggregation phenotype of *S. stutzeri* MJL19 observed in LB after several hours of growth, which is enhanced in the presence of increased NaCl (Lami et al. 2020), limits the accuracy of the fluorescence/growth normalization in liquid cultures. To reduce this problem, analyses were done in minimal medium with glucose, where cultures reach lower cell densities. The wild-type and mutant strains were inoculated in 96-well plates and fluorescence and absorbance (OD_600_) were measured during growth (Figure S4), to then normalize fluorescence values with respect to OD_600_. As shown in Fig. 6A, c-di-GMP levels increase in the wild type strain from 5 to 18–20 h of growth, followed by a slight decrease, remaining then steady from 24 h onwards. The *gacS::Km* mutant shows a different pattern, with fluorescence increase being delayed, with a peak of lower intensity than the wild type after 24 h of growth, although it ends up reaching similar values in this medium after 32 h. With respect to the EPS-deficient mutants, the Δ*bcs* strain showed a delayed fluorescence peak, reaching higher levels than the wild type and then dropping below it, whereas relative fluorescence remained lower in the other two strains, particularly in the double mutant, and no peak was observed throughout the experiment. This contrasts with the results observed in solid rich medium and suggests that the connection between EPS and c-di-GMP synthesis may be influenced by the style of growth (solid vs. liquid), aeration and perhaps additional components in LB besides NaCl, and/or by metabolic differences derived from growth in either medium that need to be further characterized. This connection was further supported by data of cellular c-di-GMP contents in MJL19, *gacS::Km* and Δ*sea* strains, obtained by LC–MS analysis as detailed in the Methods section. Results in Fig. 6B confirm the reduction in second messenger levels in these mutants compared to the wild type at 24 h of growth in minimal medium under orbital shaking.

### Identification of additional elements contributing to c-di-GMP turnover

In order to further explore the c-di-GMP signaling circuits in *S. stutzeri* MJL19, a second round of random transposon mutagenesis was carried out with mini-Tn*5*[Sm/Sp] (de Lorenzo et al. 1990), using the Δ*sea* mutant as recipient strain to identify alterations in second messenger levels. The rationale was to take advantage of the reduced extracellular matrix produced by this mutant, which we presumed would increase the efficiency of conjugation and facilitate correct plating afterwards. The Km^R^ cassette introduced in this mutant would also help counterselect donor *E. coli* cells and allow mutant selection on LB plates. This approach turned out to be successful, and over 15,000 Km^R^ Sm^R^ clones were obtained. These were pooled and the pCdrA-oriT biosensor was introduced *en masse*, to screen for clones showing changes in fluorescence, indicative of altered c-di-GMP contents. Two mutants were identified, one in which fluorescence was almost completely abolished and one showing a large increase in fluorescence, when compared with their parental Δ*sea* strain. Arbitrary PCR and sequencing of the transposon insertion sites allowed identifying the disrupted genes as *katE* (corresponding to hydroperoxidase HP-II) in the case of the reduced fluorescence mutant, and a gene (U3Q39_RS04065) encoding a protein that contains a sulfatase/alkaline phosphatase domain and is annotated as belonging to the lipoteichoic acid (LTA) synthase family in the mutant with increased fluorescence. We provisionally refer to this gene as *lsfS* (for LTA synthase family sulfatase).

**Fig. 7:**
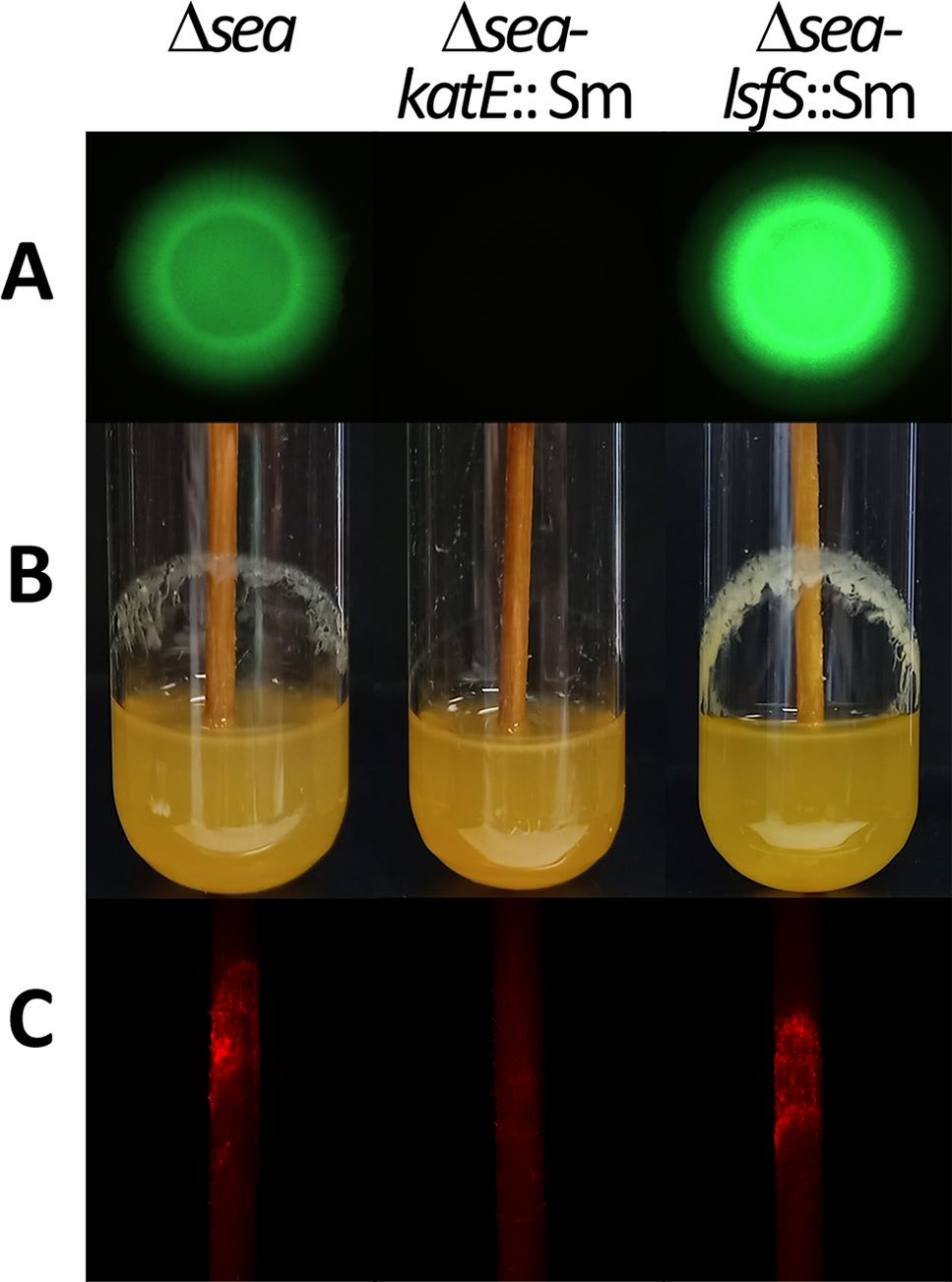
Phenotypic characterization of the Δ*sea* derivatives *katE::Sm* and *lsfS::Sm*. **A** Biosensor-based analysis of c-di-GMP contents after 24 h of growth of strains harboring pCdrA-oriT, spotted onto LB plates. **B** Biofilm formation on borosilicate glass tubes. **C** attachment to wood surface of strains tagged with miniTn*7*[P_*tac*_-mCherry]

Both mutants harboring pCdrA-oriT were cured of the plasmid, which was then reintroduced in order to confirm their phenotypes and ensure the changes in fluorescence were not due to alterations in the biosensor. As shown in Fig. 7, fluorescence due to the biosensor was completely abolished in the *katE::Sm* mutant, whereas the *lsfS::Sm* mutant showed increased fluorescence. In agreement with these observations, biofilm formation by the *katE::Sm* mutant was severely reduced, both on glass and wood surfaces. The *lsfS::Sm* mutant formed a thicker biofilm on the glass surface, but attachment to the wood surface was not significantly modified with respect to its parental strain Δ*sea*.

**Fig. 8:**
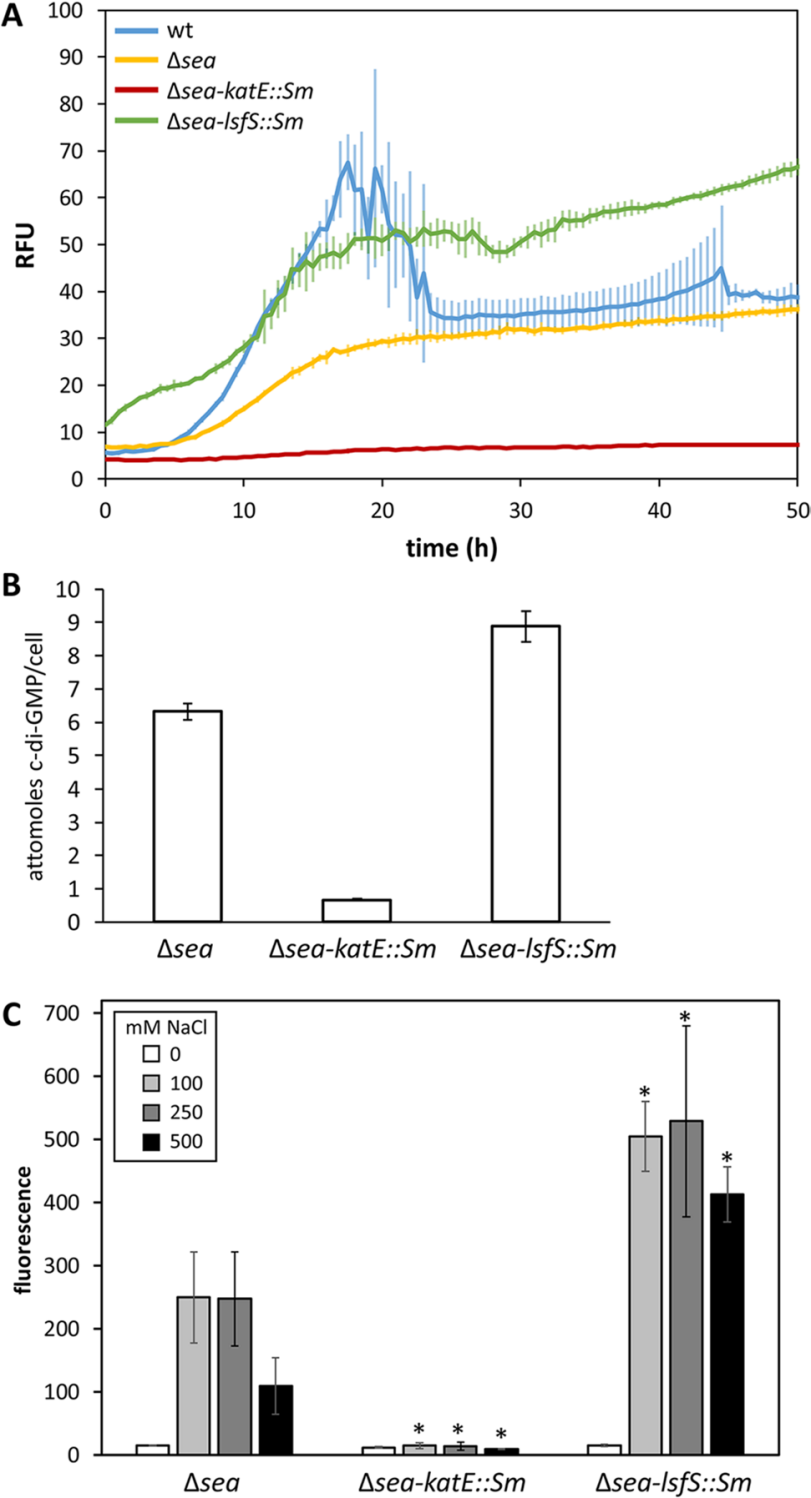
**A** Biosensor-based analysis of c-di-GMP contents in MJL19 (blue line), Δ*sea* (yellow), Δ*sea-katE::Sm* (red), and Δ*sea-lsfS::Sm* (green) during growth in liquid M9-glucose medium at 30 °C, measured in a Varioskan Lux plate reader, as indicated in Fig. 6. **B** LC–MS analysis of c-di-GMP levels in Δ*sea* and its derivatives grown in minimal medium with glucose as carbon source. Data are averages and standard deviations of two biological replicas with four technical repeats. **C** Influence of increasing NaCl concentrations on c-di-GMP levels in the Δ*sea* derivatives *lsfS::Sm* and *katE::Sm* harboring pCdrA-oriT, after 24 h of growth on modified LB. Data correspond to fluorescence units measured in a Varioskan Lux plate reader and are averages and standard deviations of five independent samples per strain. Asterisks indicate statistically significant differences with respect to the Δ*sea* parental strain in each condition tested (Student’s *t* test; *P* < 0.05)

The effect of these mutations on c-di-GMP levels was analyzed in more detail during growth in liquid M9-glucose. Results in Fig. 8A and Figure S4 show that fluorescence due to the biosensor was consistently low in the *katE::Sm* mutant, while in the *lsfS::Sm* mutant it steadily increased over time, with levels higher than those of the Δ*sea* parental strain at all timepoints. Comparison with MJL19 also revealed differences in that the reduction in fluorescence observed in the wild type after the initial peak does not take place in the *lsfS::Sm* mutant. These results were further supported by LC–MS analysis of second messenger contents in overnight cultures grown in M9-glucose (Fig. 8B).

The influence of increasing salt concentrations on c-di-GMP contents was also analyzed in these mutants after growth in solid medium. As shown in Fig. 8C, fluorescence was very low in the Δ*sea*-*katE*::Sm mutant regardless of the NaCl concentration. In contrast, the Δ*sea*-*lsfS*::Sm mutant showed increased fluorescence in all cases, except in the absence of salt.

Further confirmation of the involvement of these two elements in c-di-GMP turnover was obtained by cloning the wild-type alleles in a multicopy plasmid, which was then introduced in the respective mutants and their parental strain. The pCdrA-oriT was then incorporated and fluorescence was evaluated. Figure S5 shows that *katE* in multicopy restored fluorescence in the *katE::Sm* mutant and caused an increase in the Δ*sea* strain, while *lsfS* in multicopy had the opposite effect, indicating a reduction of c-di-GMP levels, both in the *lsfS::Sm* mutant and its parental strain.

## Discussion

In this work, we have started to elucidate the mechanisms involved in surface colonization and c-di-GMP turnover in *S. stutzeri* MJL19, a strain of particular biotechnological interest, given its plant growth-promoting characteristics in saline environments. The connections between salt concentration and second messenger and extracellular matrix production have therefore also been explored. Our results indicate that, like in other bacteria, GacS acts as a key modulator of biofilm formation in MJL19, regardless of the surface to be colonized. It functions as a positive regulator of c-di-GMP synthesis, and of expression of genes corresponding to the two identified EPS in this strain, cellulose and Sea. In other rhizosphere bacteria, such as *P. ogarae* F113, mutations in *gacS* or *gacA* lead to a hypermotile phenotype (Martínez-Granero et al. 2006). In *S. stutzeri* MJL19, the influence of GacS seems to be less pronounced, although the *gacS* mutant shows slightly increased swimming motility and surface spread on agar plates.

Analysis of expression data indicates that GacS acts as positive regulator of genes for the synthesis of both cellulose and the species-specific EPS Sea. However, biofilm formation results suggest that there is a certain diversification of functions between the two EPS, Sea being more relevant on the biotic surface, and Bcs being involved in biofilm development on the abiotic one. They also suggest that attachment to the surface and extracellular matrix production are not necessarily connected since the Δ*sea* mutant shows visible differences with the wild type in the amount of biomass associated to the toothpick that do not fully correlate with observation of fluorescently tagged cells. It should be noted that in the growth media we have tested so far, MJL19 is not very amenable to the usual biofilm assays on plastic multiwell plates using crystal violet to stain and quantify the attached biomass; biofilms are formed but the whole biomass is readily removed from the surface even upon gentle washing or careful addition of the liquid stain, making precise evaluation rather difficult.

The role of exopolysaccharides in MJL19 seems more relevant than in other *Pseudomonas* (*Stutzerimonas*) *stutzeri* strains such as XL-2, where they have been ascribed a minor effect in biofilm formation (Ding et al. 2019), with extracellular proteins being the key factors. In contrast, in strain A1501, the results are more similar to those obtained here (Shao et al. 2022), although a differentiation between biotic and abiotic surfaces has not been previously done. It should be noted that in A1501, the species-specific EPS has been referred to as *pel*, following a nomenclature similar to that of *Pseudomonas aeruginosa*. However, we consider more adequate the name used here, *sea*, to emphasize its specific nature. The more so since these are now two distinct genera.

**Fig. 9:**
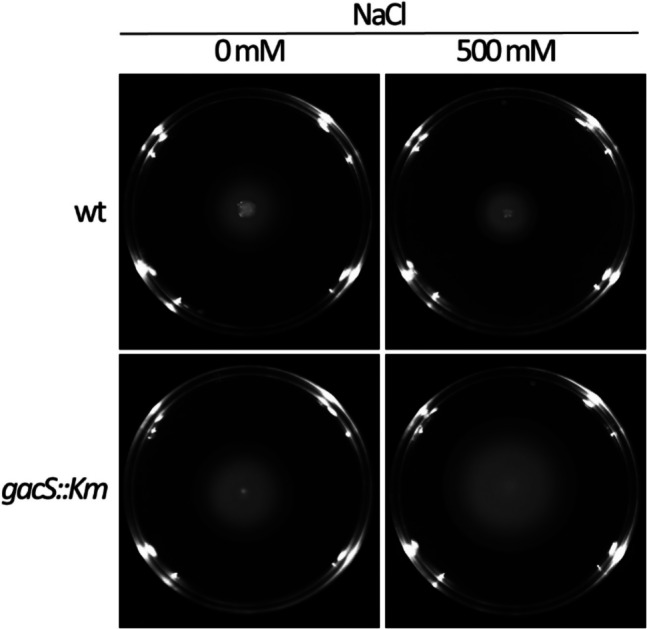
Swimming motility assay on 0.3% agar plates of modified LB containing 0 or 500 mM NaCl. Experiment was done in triplicate and a representative image is shown, obtained after 24 h of growth at 30 °C

The data obtained here support the regulatory role of GacS/GacA on c-di-GMP turnover in *S. stutzeri* MJL19, although its effect is more or less marked depending on the medium and growth conditions. This is in agreement with observations indicating an influence of the carbon source on GacS regulation in *P. chlororaphis* (Kim et al. 2014b). GacS is nonetheless essential for the response to increasing NaCl concentrations, in terms of second messenger contents, suggesting it can somehow participate in the detection of salt by *S. stutzeri*. The link between salinity and c-di-GMP via GacS is further supported by the fact that the differences between the wild type and the *gacS::Km* strains in terms of swimming motility, which in general correlates negatively with c-di-GMP levels (Hengge 2009), are more evident in the presence of 500 mM NaCl (Fig. 9). In fact, the expression of *gacS* in *P. putida* was found to be positively impacted by high concentration of NaCl (Dubern and Bloemberg 2006). In the experiments presented here, c-di-GMP levels are higher in rich medium (LB) than in defined medium (M9), and this can be mostly explained by the differences in NaCl concentration: whereas LB contains 170 mM NaCl, the concentration in M9 is around 8.5 mM, making this medium closer to modified LB without NaCl. However, the results obtained when comparing the wild type and mutant strains in these two media (M9-glucose and LB without NaCl; Fig. 5B and C) are not fully equivalent, indicating that salt is not the only factor influencing c-di-GMP levels.

Expression data, the phenotypes observed in the Δ*sea*, Δ*bcs*, and Δ*sea*-Δ*bcs*, and the results obtained with the biosensor are indicative of a regulatory interconnection between the two EPS and c-di-GMP. It seems possible that a “fabrication control” system exists, in which the cells may sense the amount of EPS being released, and if it is not in accordance with the requirements of the specific environmental situation (namely salt concentration), c-di-GMP synthesis is modified as a feedback loop to increase/decrease extracellular matrix production. This hypothesis is consistent with the data but will require further experimentation to be verified. Purification and quantification of EPS and analyses of c-di-GMP levels in the different genetic backgrounds under a wider range of environmental situations could help test this hypothesis.

We have identified additional elements altering c-di-GMP turnover in MJL19, namely the hydroperoxidase HP-II encoded by *katE*, and a putative sulfatase of the LTA synthase family encoded by a gene which we have named *lsfS*. The effect of the mutation in *katE*, which leads to a strong reduction in c-di-GMP levels, could indicate that oxidative stress functions as a signal that promotes biofilm dispersal in *S. stutzeri*, a hypothesis that will be evaluated in future research. Expression of *katE* has been reported to increase in *P. putida* when a diguanylate cyclase is overexpressed (Xiao et al. 2019), and reactive oxygen species modulate c-di-GMP levels in *P. aeruginosa* (Chua et al. 2016). The second mutation causes the opposite effect and is not easy to interpret at this point. Despite the fact that LTA is a component of the cell envelope of Gram-positive bacteria, the existence of teichoic acid-like polymers has been reported in Gram-negative species such as *Escherichia coli*, *Haemophilus influenzae*, or *Neisseria meningitidis* (Litschko et al. 2018). The protein is conserved in different species, mostly *Stutzerimonas* and *Pseudomonas* strains. It contains a sulfatase/alkaline phosphatase domain similar to that found in phosphoglycerol transferases (Figure S6) such as OpgH, which in some gram-negative bacteria participate in the synthesis of osmoregulated periplasmic glucans. Mutations affecting their production have been shown to cause pleiotropic effects, from biofilm reduction to loss of virulence (Bontemps-Gallo and Lacroix 2015). However, the similarity of this protein with OpgH and related glucan biosynthesis enzymes is limited, and genes encoding homologs of these proteins can be found elsewhere in the genome of MJL19 (loci U3Q39_006935 and U3Q39_006940). Thus, the precise role of LsfS is unclear and its connection with second messenger signaling will deserve further analysis.

Overall, we have provided here a first approach to the molecular mechanisms involved in c-di-GMP signaling and biofilm development by MJL19, a strain with promising characteristics as PGPR under saline conditions. Future work will aim at expanding this information and characterizing in further detail the elements that connect the responses to salt with the multicellular lifestyle of this bacterium with special focus in the diguanylate cyclases present in this bacterium.

## Supplementary Information

Below is the link to the electronic supplementary material.

Supplementary file1 (PDF 1707 KB)

## Data Availability

Genome data are available in GenBank with accession CP140298.1
