# Supplementary material for: Genetic basis of biofilm formation and salt adaptation in the plant-beneficial strain Stutzerimonas stutzeri MJL19
Source: Appl Microbiol Biotechnol. 2025 May 30;109(1):130. doi: 10.1007/s00253-025-13523-0 (PMC12125118; doi:10.1007/s00253-025-13523-0)
Supplement: Supplementary file 1 — Supplementary file1 (PDF 1707 KB) [file 253_2025_13523_MOESM1_ESM.pdf]

SUPPLEMENTARY MATERIAL

Genetic basis of biofilm formation and salt adaptation in the plant-beneficial strain *Stutzerimonas stutzeri* MJL19

Verónica Pérez-Padilla, María Antonia Molina-Henares, Zulema Udaondo, María Isabel Ramos González, Manuel Espinosa-Urgel*

Department of Biotechnology and Environmental Protection. Estación Experimental del Zaidín, CSIC. Profesor Albareda, 1. Granada 18008. Spain

*For correspondence. E-mail: manuel.espinosa@eez.csic.es

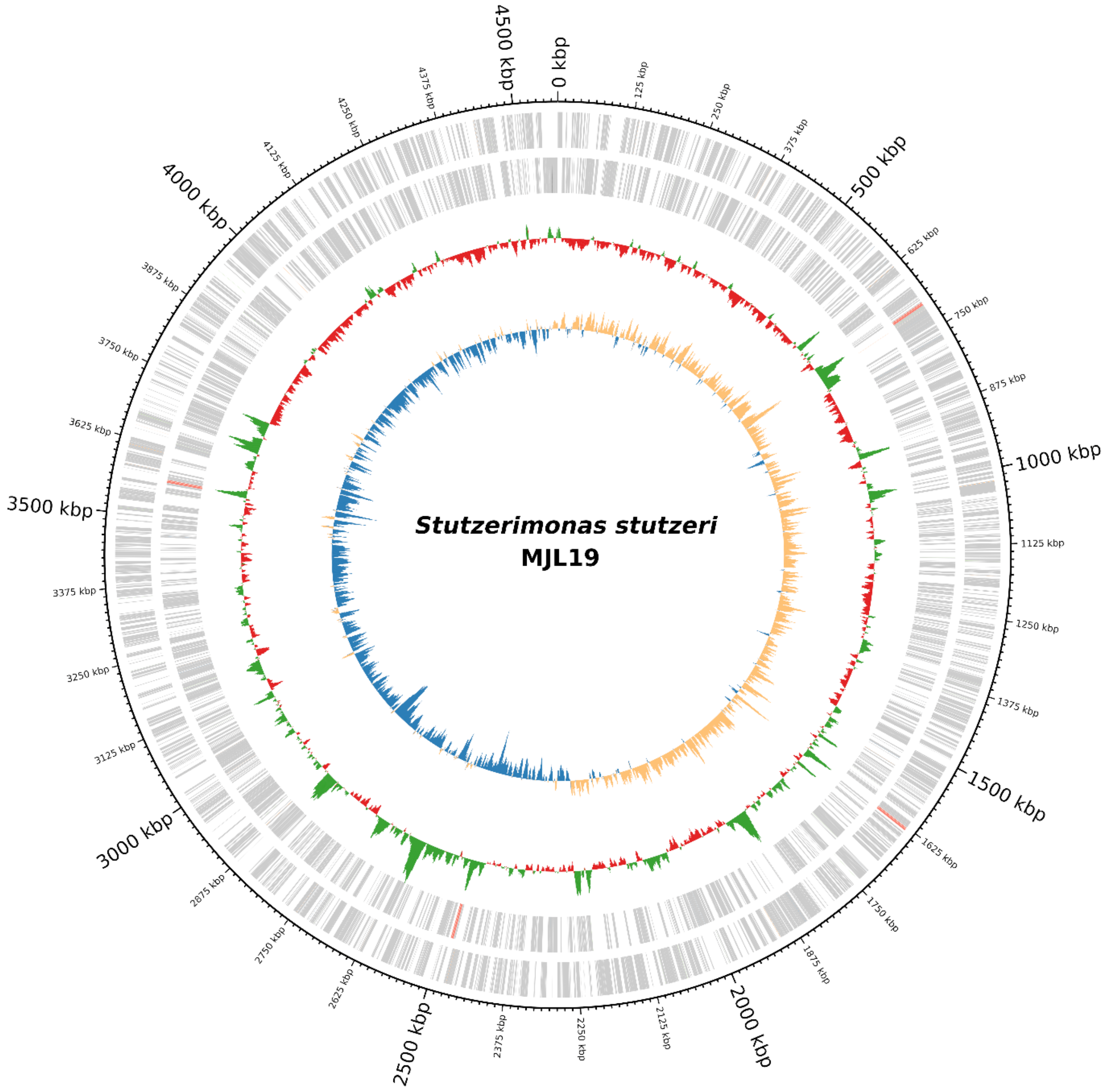

Figure S1. Full genome map of *S. stutzeri* MJL19 From outer to inner rings: 1. Genome size (black ring). 2. CDS (coding sequences). Forward and reverse strands are represented by gray bars. The four rRNA operons are highlighted in red. 3. GC content per sliding window (2500 bp). Regions with GC content higher than the genome-wide average are shown in green, while those with lower GC content are indicated in red. 4. GC skew. Positive GC skew, (indicating a higher concentration of guanine over cytosine) appears in orange, and negative GC skew (indicating a higher concentration of cytosine over guanine) is shown in blue. Map was generated using Circos via Bakta software v1.9.4 (Schwengers et al. 2021).

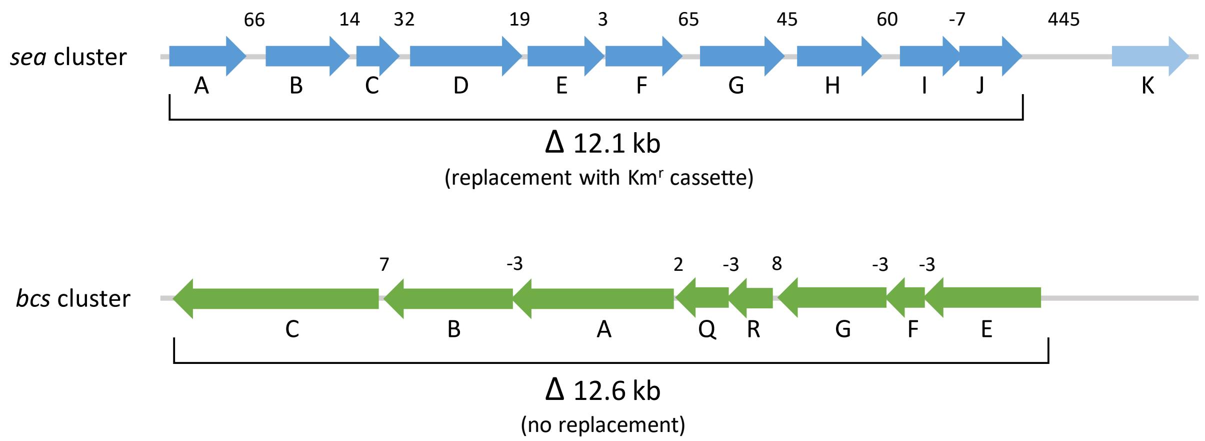

sea cluster

gene	locus	Protein accession	Annotation	Protein length
seaA	U3Q39_RS13125	WP_106443084.1	Undecaprenyl-phosphate glucose phosphotransferase	475
seaB	U3Q39_RS13130	WP_014597010.1	Low molecular weight protein-tyrosine-phosphatase	145
seaC	U3Q39_RS13135	WP_011913607.1	Polysaccharide biosynthesis tyrosine autokinase	738
seaD	U3Q39_RS13140	WP_230624785.1	Glycoside hydrolase family 5 protein	360
seaE	U3Q39_RS13145	WP_011913609.1	Polysaccharide pyruvyl transferase family protein	395
seaF	U3Q39_RS13150	WP_017245115.1	Oligosaccharide flippase family protein	410
seaG	U3Q39_RS13155	WP_013983097.1	Coenzyme F420 hydrogenase/dehydrogenase, beta subunit C-terminal domain	416
seaH	U3Q39_RS13160	WP_011913612.1	Hypothetical protein (membrane protein)	417
seaI	U3Q39_RS13165	WP_013983099.1	Glycosyltransferase family 2 protein	291
seaJ	U3Q39_RS13170	WP_011913614.1	WecB/TagA/CpsF family glycosyltransferase	255
seaK	U3Q39_RS13175	WP_017245113.1	polysaccharide biosynthesis/export family protein	366

bcs cluster

gene	locus	Protein accession	Annotation	Protein length
bcsC	U3Q39_ RS01535	WP_170799243.1	Cellulose synthase complex outer membrane protein BcsC	1152
bcsB	U3Q39_ RS01540	WP_045158003.1	Cellulose biosynthesis cyclic di-GMP-binding regulatory protein BcsB	740
bcsA	U3Q39_ RS01545	WP_170799244.1	UDP-forming cellulose synthase catalytic subunit	865
bcsQ	U3Q39_RS01550	WP_170799245.1	Cellulose biosynthesis protein BcsQ	244
bcsR	U3Q39_RS01555	WP_017244729.1	Cellulose biosynthesis protein BcsR	77
bcsG	U3Q39_RS01560	WP_170799246.1	Cellulose biosynthesis protein BcsG	541
bcsF	U3Q39_RS01565	WP_014595551.1	Cellulose biosynthesis protein BcsF	60
bcsE	U3Q39_RS01570	WP_170799247.1	Cellulose biosynthesis protein BcsE	512

Figure S2. Genetic organization of exopolysaccharide synthesis clusters in *S. stutzeri* MJL19, and functional annotation of the corresponding proteins. Numbers indicate the distance beween ORFs, in bp. The fragment deleted in each case is indicated (Δ). The double mutant was constructed on the Δ*sea* mutant.

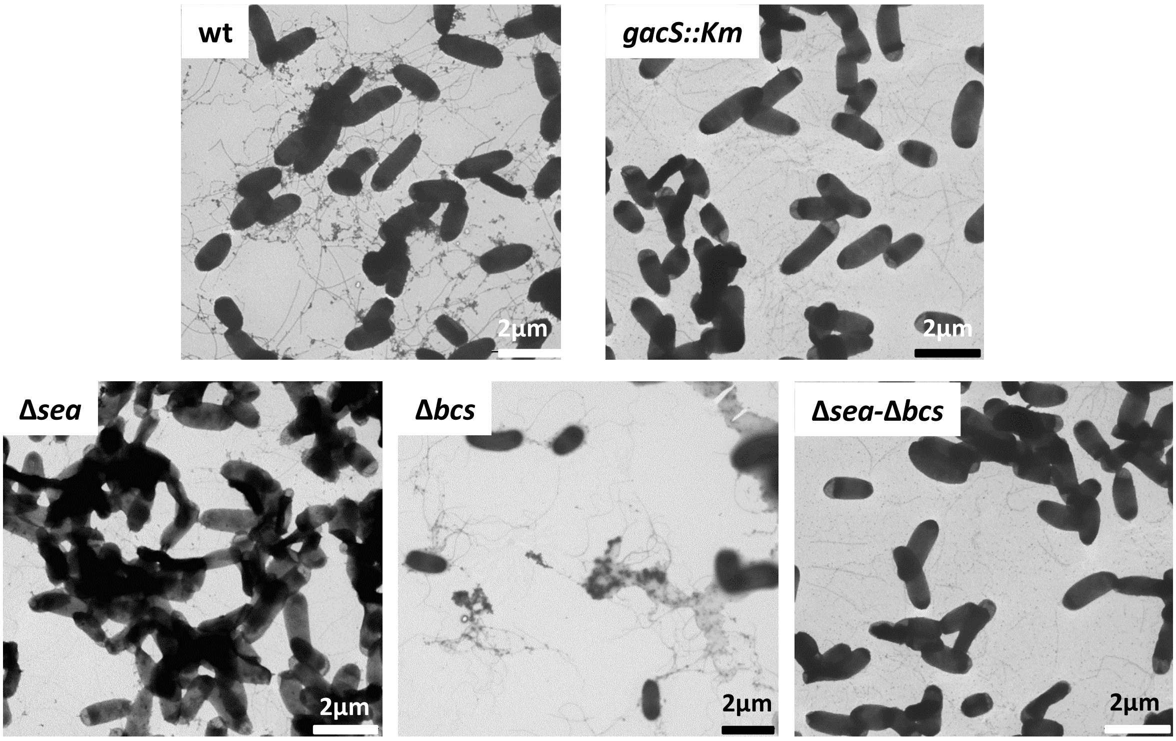

Figure S3. Transmission electron microscopy images of MJL19 and its mutant derivatives, grown overnight in liquid LB. Electron microscopy observations were carried out at the CTEM Service (EEZ-CSIC). Bacteria were recovered from overnight cultures and adsorbed on carbon-coated 200-mesh nickel grids, washed in distilled water for 1 min and negatively contrasted with 2% uranyl acetate (Polysciences Inc.). The grids were observed under a JEOL JEM-1011 transmission electron microscope at 100 kV. Images were obtained using an Olympus-SIS Megaview III capture system.

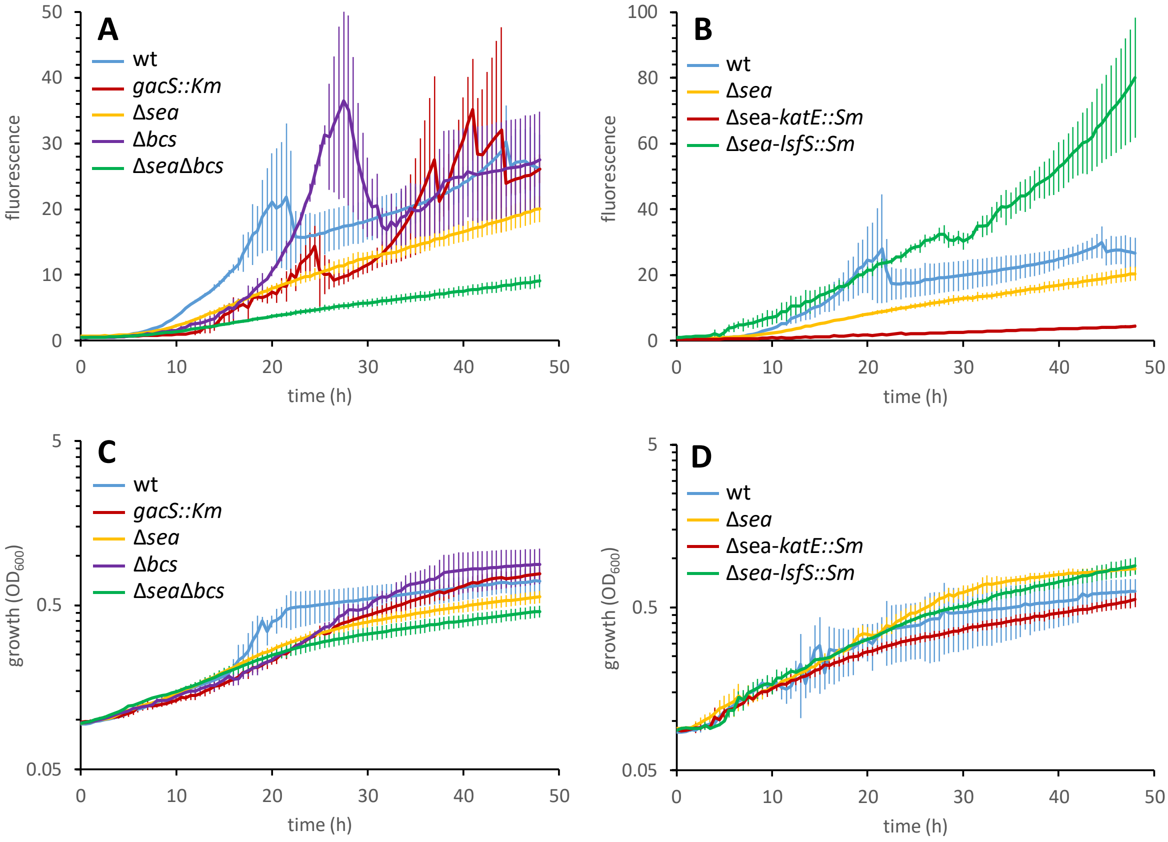

Figure S4. Fluorescence (A, B) and growth (C,D) of the different strains harboring pCdrA-oriT, measured in a Varioskan plate reader. Data are the averages and standard deviations of the raw values obtained and used for evaluating relative fluorescence in the experiments presented in Figures 6A (A normalized with respect to C) and 8A (B normalized with respect to D).

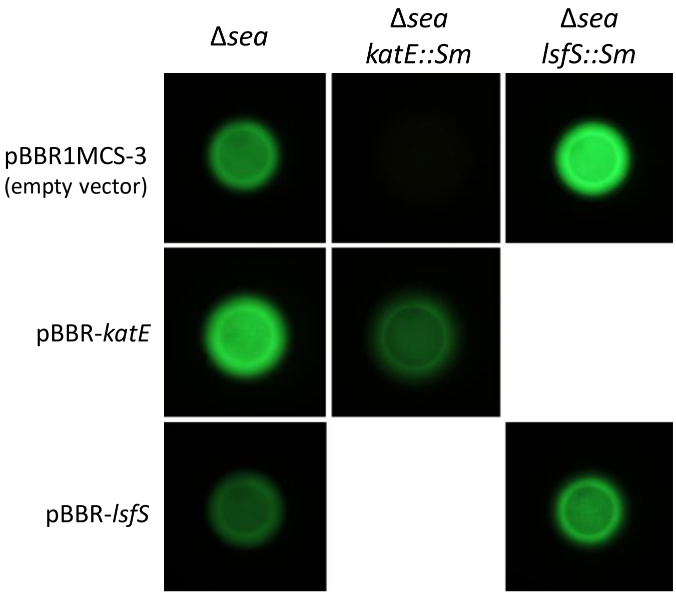

Figure S5. Analysis of c-di-GMP levels in Δ*sea* and its derivatives harboring pCdrA-oriT and the respective wild type alleles in plasmid pBBR1MCS-3, or the empty vector as control. Cultures were grown overnight, adjusted to an OD_{600} = 1 and spotted (2µL) onto LB plates with 250 mM NaCl. This concentration was chosen based on the results in Figures 5 and 8, that show a maximum of fluorescence in most strains under these conditions.

```
LsfS            1 MPFLRHAQLRYLVLLLGLWLGVFLLTRSVLLAAHWQEATAGPLELLRIYL

LsfS           51 EGTVYDLAFLAFAAVPLALYILLC-PRRLWGKRFHRLGLHLLVGLSLYLM
SO020755.1      1       MHWMLLVSLLLLLWLLVASPRLAWLK----AGL-----LSLFLL

LsfS          100 LFTALAEWLFWDEF-GVRFNFIAVDYL-VYSDEVLHNIMESYPVYF---L
SO020755.1     36 LLSA---WGLVDRLSGDGINAATLYHLRADMDGAGVSDFSGYIAVFVGML

LsfS          145 LSLISVLALLIDLALQRPVSRALDAPSLSWRGSAVALAGIASLALVSAIV
SO020755.1     83 LLSLSPLLLVRVRRFQHP------------RGGGAVFAGFVGMLLVG---

LsfS          195 VGQDFPRSSGGNAYQRELASNGPYQFFAAFRNNELDYPQFYATLQDDEIS
SO020755.1    118 -----------------IAASPLYR----------DGKRLYYQLR-----

LsfS          245 SRLRREVAEPNARFLGSDPQDIRRAIDN---PGHP--RRLNIVLVTIESL
SO020755.1    136 ------------------PVDYATVVPEYQVPQQPLQKRKNIVWIYGESL

LsfS          290 SAKYLGSFGDSR---GLTPNLDALRKQSLFFTNFYAT-GTR-TDRGLEAI
SO020755.1    168 ERTYF----DEQVFPGLMPNLRALATEAVDVRNLASTEGSGWTIAGMVAS

LsfS          335 TLSVPPT--PG-RSIVKRIGRESGFA-SLGQQLGAQGYDSVFLYGGRGYF
SO020755.1    214 MCGVPLTTAPGDENSMDRMGMFLPEARCLGDYLKDQGYRNHYVGGADASF

LsfS          381 DNMSAFFGGNGYRVV-DQSSVKEADIRFK--NAWGMADEDLYTLALREAD
SO020755.1    264 AGKGRFLSSHGFDVVHDVHHFHDKGVAQKHFSAWGVHDDVLLDDAWDSFQ

LsfS          428 ADHAAGTPFLLQLMTTSNHRPYTY-PDG-RI---DIPSGE-GREGAVKYT
SO020755.1    314 TLSRAGQPFMLTTLTMDTHHPAGHLPLACKGQHYDSALGDIGLLHAIKCS

LsfS          468 DFAIGQFLEQARRKPWFASTLFVFVADHTAGSAGSQDLPVANYHIPLFVY
SO020755.1    360 DRLIGELVARIRNSRYGKNTIIVIASDHLAMPNDLSDVLAKQKRENLLLF

LsfS          518 APGLVEPGE-FSGVASQIDIAPTLLGLLNMDYVSTFFGRNLLREDAAPGR
SO020755.1    410 LGKDIAPQQVVTRAGSTLDSGATLLQLLEPGMRTLGFGRSLLASDAPPSA

LsfS          567 ALI-----G-NYQH-LG----LFDGRDLAILSPRLRVRRHDEALRSSRES
SO020755.1    460 SVAASRDSGKDYPRYLAYARTLWTGR--STRM--LRINGNGDVVVGVQQV

LsfS          606 EAPLSDPLVQRD------IAYYQGASHDY---H-QGLLS-----------
SO020755.1    506 RPPV---LLEYDKDTNLKTVYLENTSRQFDRTHSKGTLAYVDRCTAFEDG

LsfS          639 -----WQALTPHHGHLSQR
SO020755.1    553 SADGDWCALVVDR-HQSMKLYRDPDLTRGIAVDAPLEATQQGPRPRVRQP

SO020755.1    602 IMLTQEARKTDAGRYMLELYAKRRPTRAFWVEAVSSERKVVLAQQWVVPD

SO020755.1    652 AAGRIRMPVGLEHAVEDLEIRAWLDYTEDVSVDDLALVKDIPVADRS
```

Figure S6. Pairwise alignment between LsfS and phosphoglycerol transferase I from *Xanthomonas citri* pv. *fuscans*. Identical and conserved residues are highlighted in yellow and grey, respectively. The approximate region corresponding to the sulfatase/alkaline phosphatase domain is boxed.

Table S1. Primers used in this study. Restriction sites inserted in the primer for cloning are underlined.

Name	Sequence (5'→3')	Comment
**Uni**<br>**Rev**	GTTTTCCCAGTCACGACG<br>GCGGATAACAATTTCACACAG	Sequencing of fragments cloned in pCR™-Blunt II-TOPO®
**bcsUpF**<br>**bcspR**<br>**bcsDwF**<br>**bcsDwR**	ATTGCGGCCGCTGATCGGGATCTGGTTGTTC<br>ACTGGGTCGGTCAGAAGCTTAAGTCACCTGCGGCGGTTT<br>AAGCTTCTGACCGACCCAGTTCAAGCGGGCGCAGTTATAC<br>ATTGCGGCCGCGGTTTCCAGGCTGGTGTAGA	*bcs* null mutant construction
**seaUpF**<br>**seaUpR**<br>**seaDwF**<br>**seaDwR**	ATTGCGGCCGCCAGCACCCGCTTGAGCTCCT<br>TGTACTGATCGACGCACGCTCACAAGGCTCCATCCTCAGCTC<br>GAGCTGAGGATGGAGCCTTGTCAGGACAGCTGACGCATTCATC<br>ATTGCGGCCGCTCACGCTTGAGGGTCAGACC	*sea* null mutant construction
**bcsintFw**<br>**bcsintRv**	TCGATGGCGAGCGACATCAG<br>GGGATTGCCGAACAACGCAT	Internal primers for sequencing and checking the deletion in Δ*bcs* mutant
**seasecFw**<br>**seasecRv**	ATTATCCGCGTCCTGCTTAG<br>TGCCTGAGGCGTTTGAATTG	Internal primers for sequencing and checking the deletion in Δ*sea* mutant
**ARB1A**<br>**ARB1D**<br>**TNEXT2**<br>**TNTETU**	CCACGCGTCGACTAGTACNNNNNNNNNNNGATAT<br>GCCACCCGTCGACTAGTACGNNNNNNNNNNGATT<br>CTTTATTGATTCCATTTTACACT<br>AGCATAAAGCTTGCTCAATC	First round of arbitrary PCR
**ARB2A**<br>**ARB2D**<br>**TNINT**<br>**TNINT7**<br>**TNTCO**<br>**TNINT-Sm**	CCACGCGTCGACTAGTAC<br>GCCACCCGTCGACTAGTACG<br>GACCTGCAGGCATGCAAGCTCGGC<br>CGACCTGCAGGCATGCAAGC<br>CGCACTTGTGTATAAGAGTCAG<br>CGGGAATTCGTCGACAAGCT	Second round of arbitrary PCR
**fps158**<br>**rps743**	GTGGGGGACAACGTTTC<br>CACCTCAGTGTCAGTATTAGC	Specific primers for *S. stutzeri* (Bennasar, et al. 1998)
**Km1Fw**<br>**Km1Rev**	GAGCCATATTCAACGGGAAACG<br>CTCACCGAGGCAGTTCCATAG	Detection of miniT5[km1]
**miniTn*5*-SmF**<br>**miniTn*5*-SmR**	ATGAGGGAAGCGGTGATCG<br>TTATTTGCCGACTACCTTGGTG	Detection of miniT5[Sm/Sp]
**gacSF**<br>**gacSR**	GGATCCTGATCGAAGGTGCGG<br>TCTAGAGTCGGTCAGCCGTTGG	Complementation of *gacS* mutation
**katEF**<br>**katER**	CCCGGGCGATCACGGGCGAACCTTGT<br>GAGCTCGGCGCCTCATCCGTGTG	Complementation of *kat*E mutation
**lsfSF**<br>**lsfSR**	CCCGGGGAGGATGTCACGGTTGTCTTC<br>GAGCTCGTCGGATAGAGCAGACATCGG	Complementation of *lsfS* mutation
**q16S-F**<br>**q16S-R**	GCGGATGAGTGGAGCTTGC<br>GCTAATCCGACCTAGGCTCATCT	qPCR - 16S rRNA
**qSea-F**<br>**qSea-R**	CCCTTGAATATGCTCACCCGAGTT<br>TATAGATGCCGACGGTCTGGAAGA	qPCR - cluster *sea*
**qbcs-F**<br>**qbcs-R**	CCTGCATTGGATCATGGCGGAAA<br>CGCTGCAGCATCGCTTCGAAAT	qPCR - cluster *bcs*
**oriT-BspEI-Fw**<br>**oriT-BspEI-Rev**	TCCGGAGCAGATTGTACTGAGAGTGC<br>TCCGGACATCTGTGCGGTATTTCA	amplification and cloning of oriT into pCdrA::*gfp*^{c}
**Tn7RR109**	CAGCATAACTGGACTGATTTCAG	Confirmation of miniTn*7* insertion
**P.s.glmS3'**	AACCTGGCCAAGTCGGTTAC	

References

Bennasar A, Guasp C, Tesar M, Lalucat J. (1998) Genetic relationships among *Pseudomonas stutzer*i strains based on molecular typing methods. J Appl Microbiol. 85:643-656. doi: 10.1111/j.1365-2672.1998.00572.x.

Schwengers O., Jelonek L., Dieckmann M. A., Beyvers S., Blom J., Goesmann A. (2021). Bakta: rapid and standardized annotation of bacterial genomes via alignment-free sequence identification. Microb Genom 7:000685. doi: 10.1099/mgen.0.000685
